# Supplementary material for: Genetic mapping and evolutionary analysis of human-expanded cognitive networks
Source: Nat Commun. 2019 Oct 24;10:4839. doi: 10.1038/s41467-019-12764-8 (PMC6813316; doi:10.1038/s41467-019-12764-8)
Supplement: Supplementary file 1 — Supplementary Information [file 41467_2019_12764_MOESM1_ESM.pdf]

Supplementary Information

Genetic mapping and evolutionary analysis of human-expanded cognitive networks

Wei et al.

Table of Contents

Supplementary Methods 3

MRI data 3

MRI processing 4

Cortical expansion 5

BB-38 chimpanzee-human atlas 5

Human transcription data from AHBA 6

Transcription data of the human, chimpanzee, and macaque 7

HAR identification using brain-related Hi-C and eQTL 8

Functional networks 8

Top genes differentiating genes of the default-mode network 9

UK Biobank GWAS on DMN functional activity 10

Cortical vulnerability in psychiatric disorders 13

Supplementary Note 1. Alternative identifications of HAR genes 15

Supplementary Note 2. Alternative identifications of DMN genes 16

Supplementary Note 3. Examination of the potential effects of tissue sample number 17

Supplementary Note 4. Alternative cortical parcellation atlases 18

Supplementary Note 5. Alternative functional network divisions 19

Supplementary Note 6. Directly mapping AHBA tissue samples to functional networks 19

Supplementary Note 7. Alternative parameters in processing 20

Supplementary Note 8. HAR-BRAIN gene expression in subcortical regions 22

Supplementary Note 9. Discussion on evolutionarily cortical expansion 22

Supplementary Tables 24

Supplementary Figures 33

Supplementary References 47

Supplementary Methods

MRI data

We calculated cortical expansion using in vivo MRI data from 29 chimpanzees (*Pan troglodytes*; age: 30.2 ± 12.6, all female) and 30 humans (*Homo sapiens*; age: 29.8 ± 3.2 years; all female). Chimpanzees were accommodated at the Yerkes National Primate Research Center (YNPRC) in Atlanta, Georgia. All procedures were implemented under protocols approved by the YNPRC and the Emory University Institutional Animal Care and Use Committee (IACUC, approval #: YER-2001206). No new MRI data was acquired for this study. All chimpanzee MRIs were obtained from a data archive of scans obtained prior to the 2015 implementation of U.S. Fish and Wildlife Service and National Institutes of Health regulations governing research with chimpanzees. All the scans reported in this publication were completed by the end of 2012. All MRI scans are part of the National Chimpanzee Brain Resource (http://www.chimpanzeebrain.org).

Chimpanzee MRI scans were collected from chimpanzees under anesthesia with isoflurane (1%) (protocols described in detail in [1]). Chimpanzees were immobilized with ketamine (2-6 mg/kg). Constant observation was given by veterinary staff for chimpanzees before, during, and after scanning. Head motion was minimized using foam cushion and elastic straps. A standard circularly polarized (CP) birdcage coil was used because they did not fit the standard phase-array coil designed for humans. T1-weighted MPRAGE imaging of all chimpanzees was obtained on two Siemens 3T Trio Tim Scanners (Siemens Medical System, Malvern, PA) with the following parameters: slice thickness = 0.8 mm, voxel size = 0.8 × 0.8 × 0.8 mm, TR = 2,600 ms, TE = 3.06 ms, matrix size = 256 × 256 × 192, FOV = 224 × 224, flip angle = 8 degree, scanning time = 16 min.

Data of human subjects were randomly selected from the Q3 data release of the Human Connectome Project (HCP, http://www.humanconnectomeproject.org/)[2]. Human MRI scans were acquired on a Siemens Skyra 3T scanner (Siemens Medical System, Malvern, PA) with a customized SC72 gradient insert[2]. T1-weighted MPRAGE images were collected with following scanning parameters: voxel size = 0.7 × 0.7 × 0.7 mm, TR = 2400 ms, TE = 2.14 ms, matrix = 320, 256 sagittal slices, slice thickness = 0.7 mm, FOV = 224 × 224, flip angle = 8 degree, scanning time = 7:40 min.

MRI processing

Chimpanzee and human T1-weighted MRI data were processed in the FreeSurfer software[3, 4] for brain tissue segmentation and cortical mantle reconstruction. Inspired by a recent human-chimpanzee morphological comparison based on FreeSurfer[5], chimpanzee-to-human cortical expansion was computed as follows. Individual reconstructed pial surfaces of both chimpanzees and humans were re-meshed to an identical number of vertices. FreeSurfer inflated the white matter – gray matter surface of each subject to a sphere, and registered the sphere to the standard reference ($FS_HOME/average/?h.average.curvature.filled.buckner40.tif) by aligning the curvature data, resulting in a surface file of the registered sphere. Next, for each vertex *i* (the target vertex) on the registered sphere of the fsaverage, we extracted the face (i.e., a triangle formed by three vertices) comprising the location of vertex *i* on the registered sphere of each subject. To do this, we used Barycentric coordinates:

$$\mathbf{P}_i = [x_i, y_i, z_i] \qquad [1]$$

$$\begin{bmatrix} u \\ v \\ w \end{bmatrix} \times \begin{bmatrix} \mathbf{P}_1, 1 \\ \mathbf{P}_2, 1 \\ \mathbf{P}_3, 1 \end{bmatrix} = [\mathbf{P}_t, 1] \qquad [2]$$

where $\mathbf{P}_i$ is a vector of coordinates of a vertex *i*, $\mathbf{P}_1$, $\mathbf{P}_2$, $\mathbf{P}_3$ are coordinates of three vertices forming a face, $\mathbf{P}_t$ contains coordinates of the target vertex, and *u*, *v*, and *w* are numbers following $u + v + w = 1$. Given a target vertex on the registered sphere of the fsaverage template, we calculated *u*, *v*, *w* for each face on subjects' registered sphere and selected the face when all $u, v, w > 0$, which indicates that the location of the target vertex is comprised in the selected face. Using the calculated *u*, *v*, *w*, we generated coordinates of a new vertex within the selected face on subject's pial surface according to equation [2]. This way, pial surfaces of all chimpanzee and human subjects were re-meshed, resulting in spatially matched vertices across all subjects.

The re-meshed pial surfaces of all subjects were examined manually for both chimpanzee and human parcellations (i.e., 114- or 219-region subdivision of the Desikan Killiany atlas[6, 7]). An inconsistency of parcellation of the cuneus in the chimpanzees was noticed due to an inaccuracy in the registration process. Therefore, the corresponding annotation files were manually corrected for the parcellation of the cuneus lobe for all chimpanzees (according to the criteria that cuneus is bounded anteriorly by the parieto-occipital sulcus and inferiorly by the calcarine sulcus[8]). As FreeSurfer failed to properly parcellate the parahippocampal gyrus and entorhinal cortex in five of the 29 chimpanzees, we excluded these

two regions in all cortical expansion-relevant analyses. Note that we further validated the expansion and gene expression results by using the BB-38 homologous chimpanzee-human cortical atlas[9] (see below).

Cortical expansion

Cortical expansion was computed based on the reconstructed pial surfaces of both chimpanzees and humans. First, the area was calculated for each face within the re-meshed pial surface of each subject. A regional-level cortical surface area (S_i) was computed by summing up face areas within each cortical region, for all regions of the atlas (DK-114[6, 7]; also 219-region subdivision [DK-219] and the chimpanzee-human cytoarchitectonic cortical atlas [BB-38] for validation purposes, see below). Normalized cortical area was then obtained by dividing the regional area by the area of the whole cortex. Two-sided two-sample *t*-test was used to examine the between-species difference of the normalized surface area. Then, cortical expansion between every pair of chimpanzee and human subjects was calculated based on both the raw and normalized cortical surface area by

$$E_{i,j} = \frac{S_{\mathrm{human},i} - S_{\mathrm{chimp},j}}{S_{\mathrm{chimp},j}} \qquad [3]$$

with $E_{i,j}$ denoting the expansion from chimpanzee *j* to human *i*. They yielded a total of 870 (i.e., 29 × 30) chimpanzee-to-human expansion maps. Finally, a group-level region-wise cortical expansion map was made by averaging among the 870 chimpanzee-to-human comparisons.

BB-38 chimpanzee-human atlas

Results regarding the chimpanzee-human cortical expansion were validated using the BB-38 atlas that describes homologous cortical areas across the two species. In 1950, von Bonin and Bailey reported this cortical atlas[10], assessing cytoarchitectural homologies of the chimpanzee cortex based on the human cortical atlas of von Economo and Koskinas[11]. They provided detailed descriptions of 44 cytoarchitecturally distinct cortical regions of the chimpanzee brain, and how these regions relate to the human brain in terms of cytoarchitectural properties such as cortical layer thickness, neuronal cell type, size, and density[10]. Using a FreeSurfer version of the Von Economo - Koskinas atlas[8], we created the BB-38 chimpanzee-specific and homologous BB-38 human-specific cortical atlas, with 6 of the original 44 regions (PCop, FCop, FDgamma, PA, PD, and TC) excluded because they could not be properly segmented in FreeSurfer (as they were too narrow, located within a sulcus, or embedded within another

cortical region). Segmentation and atlas building followed the same procedures as described in prior literatures[8].

Human transcription data from AHBA

Microarray gene expression dataset was collected from postmortem brains of six human donors and downloaded from the Allen Human Brain Atlas (AHBA) (http://human.brain-map.org/static/download). Subjects had no history of neuropsychiatric or neuropathological disorders (demographics are tabulated in Supplementary Table 10). Tissue samples were collected for microarray analysis by either manual macrodissection for large regions (cortical and subcortical structures) or by laser-based microdissection for smaller regions (subcortical and brainstem areas)[12]. An average of 466 samples (left hemisphere) were obtained from four donor brains (466 ± 72.6 samples from H0351.1009, H0351.1012, H0351.1015, and H0351.1016). The remaining two donor brains (H0351.2001 and H0351.2002) supplied 946 and 893 samples, respectively, covering both hemispheres. Cortical samples from the left hemisphere were included in the current study[13]. For each sample, RNA isolation, quantification, normalization, and quality control were performed. Microarray analysis was conducted by Beckman Coulter Genomics company (for details, see "Technical White Paper: Microarray Survey" in http://help.brain-map.org/display/humanbrain/Documentation). The normalized expression levels of 58,692 probes representing 20,737 genes were subsequently obtained. We updated gene symbols by replacing the previous and alias gene symbols by the approved symbols from the HUGO Gene Nomenclature Committee (HGNC) database (http://biomart.genenames.org/). For each donor brain, expressions of probes corresponding to the same gene symbol were averaged, resulting in an array containing 20,734 expression levels for each sample. Gene expression levels were further normalized within each sample by dividing expression values by the mean expression of the sample.

Next, tissue samples were spatially mapped to FreeSurfer cortical regions in order to obtain region-wise gene expression profiles, using an approach similar to the method proposed in a prior study[14]. First, the sample annotation data, including the Montreal Neurological Institute (MNI) coordinates and the structure type of each sample, was extracted in the dataset downloaded from AHBA website. Samples annotated outside the left hemisphere of cerebral cortex were excluded. Second, FreeSurfer software was applied to process the MNI-152 template for brain tissue segmentation and cortical mantle reconstruction[3]. The reconstructed cortical mantle was parcellated into the distinct cortical regions of the atlas used (i.e., DK-114

with 57 regions per hemisphere based on the Cammoun subdivision of the Desikan-Killiany atlas[4, 6, 7]). A finer subdivision of the Desikan-Killiany atlas containing 219 regions (111 regions on the left hemisphere) was used for a validation, as well as the cytoarchitectonic chimpanzee-human homologous BB-38 atlas (see below). Third, for each sample in the AHBA data, the nearest voxel in the MNI 152 template was searched, according to the Euclidean distance computed between MNI coordinates of the AHBA sample and all gray matter voxels in the MNI 152 template. Each sample was assigned to a cortical region based on the nearest gray matter voxel. A distance threshold of 2 mm was used to exclude inaccurate assignments of cortical regions (also 1 mm and 3 mm were examined for validation). The assignment was manually verified to ensure that no subcortical sample was included. Finally, for each donor brain, gene expression profiles of samples belonging to the same cortical region were averaged, resulting in a 6 × 57 × 20,734 data matrix (i.e., donors × cortical regions × genes). Within each donor, gene expressions were normalized to *Z* scores across all cortical regions per gene. The normalized gene expression profiles were averaged across 6 donor brains to obtain a group-level gene expression matrix of a size of 57 × 20,734.

Transcription data of the human, chimpanzee, and macaque

Cortical transcription data of the human (Homo sapiens), chimpanzee (Pan troglodytes), and macaque (Macaca mulatta) were obtained from the PsychENCODE database (http://evolution.psychencode.org/)[15]. The PsychENCODE database provides expression levels of 16,463 genes for 16 homologous brain locations (10 cortical, 5 subcortical, 1 limbic) in humans (6 subjects), chimpanzees (5 subjects), and macaques (5 subjects; Supplementary Table 11)[15]. The age of specimens of all three species were in their respective young to early middle adulthood, and sex was matched across species. No signs of neuropathological abnormalities were reported in any of the specimens from the three species, as reported by Sousa et al[15]. The expression levels of genes were quantified by RPKM (reads per kilobase of exon model per million mapped reads). Batch effects were corrected using R package ComBat[16] to normalize the expression values. We additionally performed *Z* score transformation across brain areas within each individual to quantify gene expressions within the same scale, as suggested by Arnatkevičiūtė et al. (2019)[17]. We used the data from the ten cortical regions out of the total 16 brain regions, which included six regions of the higher-order cognitive networks (e.g., dorsolateral prefrontal, inferior parietal, middle frontal, orbital frontal, superior temporal, and ventral lateral prefrontal cortex) and four regions of the primary

networks (e.g., primary auditory, primary visual, primary somatosensory, and primary motor cortex). Normalized gene expression data was averaged across individual brains to obtain a group-level gene expression matrix of size of 11 × 16,463 for each of the three species.

HAR identification using brain-related Hi-C and eQTL

The set of HAR genes as used in the main text was obtained from the study of Doan et al.[18], as those where HARs locate within the introns, within or near (less than 1kb) 5′ and 3′ UTRs, or are the closest flanking gene that was less than 2.1mb away, (with 70% being less than 500kb away)[18]. An alternative approach to identify HAR genes can be gene mapping based on brain related chromatin interaction or eQTL. PsychENCODE provides variety of functional genomic datasets (http://resource.psychencode.org/)[19]. We obtained significant eQTLs and enhancer-promoter (gene) linkages based on HiC in prefrontal cortex from http://resource.psychencode.org/. In both dataset, genes were provided by ensemble gene ID. We filtered on protein coding genes based on Ensembl v92 GRCh37. HAR regions that overlapped with significant eQTLs were mapped to the genes whose expression is potentially affected by the SNPs in the HAR region. Similarly, enhancer regions were overlapped with HAR regions and mapped to genes whose promoter is interacting with the potential enhancer.

Functional networks

Resting-state functional networks were obtained from the Yeo 7-network atlas[20]. The Yeo atlas contains a parcellation map of 7 large-scale functional resting-state networks, including the visual, somatomotor, dorsal attention, ventral attention (VAN; also referred to as salience), limbic, frontoparietal (FPN; also referred to as central executive), and default mode network (DMN). An annotation file of the 7 functional networks was included for the fsaverage subject in the FreeSurfer Software package (https://surfer.nmr.mgh.harvard.edu/fswiki/CorticalParcellation_Yeo2011). We assigned each cortical region in the Desikan-Killiany atlas[6, 7] (BB-38 atlas for validation purposes) to one of the 7 functional networks. For this, the surface-based annotation was translated to a 3D brain volume in volumetric space, in which each gray matter voxel was assigned a network label. Next, for each region in the Desikan-Killiany atlas, we computed the ratio of voxels that belonged to each of the 7 functional networks. Using majority vote, the label of the functional network corresponding to the majority of voxels was then assigned to that region. The regional mapped functional network assignment is shown in Fig. 2b in the main text. In a validation

analysis, DMN regions were additionally cross-referenced to the definition of the DMN by Raichle et al for a validation[21], resulting in 14 regions forming the DMN (Supplementary Figure 10). A finer parcellation of the Yeo atlas in 17 networks[20] was also used for validation purposes (Supplementary Figure 11).

Top genes differentiating genes of the default-mode network

Two-sided two-sample *t* tests were performed for each of 20,734 AHBA genes to examine the difference in gene expression between regions of the DMN and the rest of the brain. Genes showing the top 200 largest *t* scores were selected as the DMN genes. We alternatively examined the top 53 ($p < 0.05$, partial Bonferroni corrected) and 469 genes ($p < 0.01$, not corrected) for validation purposes. Notably, because expression levels of genes were not independent, partial Bonferroni correction[22, 23, 24] was used in the current study, with the threshold determined based on the number of principle components ($n = 36$) explaining 95% of the total variance of the gene expression of all genes using principal component analysis (PCA), resulting in an α-threshold < 0.05/36 = 0.0014.

We calculated the ratio of genes within the DMN genes overlapping with the 1,711 HAR genes. For statistical evaluation, we tested the enrichment of HAR genes for DMN genes using hypergeometric test. To examine whether HAR genes were specifically enriched for DMN genes, a permutation analysis was performed. In each of the 10,000 permutations, functional network labels were shuffled and top 200 genes showing the largest expression differences between the reshuffled DMN and other regions were selected. The proportion of HAR genes in these top genes was computed to generate a null distribution. The original ratio was then compared to the null distribution to obtain the proportion of random permutations that exceeded the original ratio, and a *p* value was generated accordingly.

Gene-enrichment analysis was performed for the set of top DMN genes by means of hypergeometric test to examine whether DMN genes were enriched for the predefined gene sets in three functional categories, including biological process, molecular function, and cellular component, based on the Gene Ontology (GO)[25]. For each of the predefined gene sets, a *p*-value was calculated based on the number of genes present in both the predefined set and the DMN gene set. The resulting *p*-values were corrected for multiple testing through FDR with $q < 0.05$.

UK Biobank GWAS on DMN functional activity

The UK Biobank study protocol was approved by the National Research Ethics Service Committee North West Haydock (reference 11/NW/0382), and all procedures were conducted in accordance with the ethical principles for medical research declared in the World Medical Association Declaration of Helsinki. Access to the UK Biobank data was obtained under the UK Biobank application number 16406. All participants were recruited by invitation letters that were sent out to approximately 9.2 million individuals between 37 - 72 years living within 25 miles distance from one of the 22 study assessment centers and were registered with the National Health Service (NHS). Data collected included genotype data ascertained from blood samples and a wide array of phenotypic data, such as registry-based phenotypic information, extensive self-reported baseline data collected by questionnaire and brain imaging, among others.

Genetic data and processing

Genome-wide association study (GWAS) was performed based on a cohort of 6,899 participants from UK Biobank (http://www.ukbiobank.ac.uk)[26]. Details on all subsequently described genetic procedures and quality control are described previously (Savage et al.[27]) and include the following steps:

Imputed genotype data were obtained from the second release by UK Biobank (July 2017), including 92,693,895 genetic variants in 487,442 individuals[28]. Genotyping was divided over 106 batches using two custom Affymetrix genotyping platforms (UK BiLEVE Axiom array *n* ~= 50,000; UK Biobank Axiom array *n* ~= 450,000). Quality control of the genotype data was performed locally by the UK Biobank (details available at [26]). Genotypes were imputed using a combination of two reference panels: 1) a merged reference panel that included the UK10K haplotype panel and 2) the 1000 Genomes reference panel. In addition, the genotype data was imputed using the Haplotype Reference Consortium (HRC) reference panel[29]. If variants were imputed in both panels, the HRC imputation was retained.

In the current GWAS, only individuals of European ancestry were included, defined by projecting ancestry principal components from the 1000 Genomes reference populations[30] onto the called genotypes available in UK Biobank, and classifying individuals into their closest ancestral population according to the minimum Mahalanobis distance from the projected principal component scores[31]. We excluded subjects with a Mahalanobis distance > 6 standard deviation (SD) from their empirically assigned population. Additional filtering of individuals was based on UKB-provided information on genomic relatedness (subjects with most inferred

relatives, 3rd degree or closer, were removed until no related subjects were present), discordant sex, sex aneuploidy, missing phenotype or covariate data, and withdrawn consent.

Imputed variants were converted to hard calls at a certainty threshold of 0.9, filtering by an imputation INFO threshold of < 0.9 and excluding multi-allelic SNPs, indels, SNPs without a unique rsID, and SNPs with minor allele frequency (MAF) < 0.005, resulting in a total of 9,213,044 remaining SNPs for analysis. To correct for population stratification, we computed European-specific principal components based on a set of 145,432 independent (r^2 < 0.1) autosomal SNPs with MAF > 0.01 and INFO = 1 using FlashPCA2[32].

Genome-wide analysis was performed on the amplitude of fMRI time series of the 25 spatial maps identified using independent component analysis (ICA; referred to as "NETMAT amplitudes 25" in https://www.fmrib.ox.ac.uk/ukbiobank/; for a detailed description, see [33, 34]). We specifically selected 7 ICA-based brain maps that resemble the functional networks used in the current study, including maps derived from the component #1 (DMN), #2 (VN), #3 (VAN), #5 (FPN.R), #6 (FPN.L), #10 (SMN), and #14 (LN), with particular interests in the DMN (referred to as "NETMAT amplitude 25 (01)" in http://big.stats.ox.ac.uk/). Moreover, to correct for the whole-brain effect, we divided the amplitude of the component #1 by the sum of the amplitudes of all available ICA components and performed an additional GWAS on this phenotype. GWAS was conducted in PLINK v2.0[35] using an additive linear regression model and controlling for covariates of age, sex, twenty European-based ancestry principal components, and total brain volume (computed for each individual as the sum of volume of grey and white matter provided by FreeSurfer[3]).

Details on all subsequently described genetic procedures and quality control were taken from the study of Savage and colleagues[27]: all files were checked for data integrity and accuracy. SNPs were filtered from further analysis if they met any of the following criteria: imputation quality (INFO/R^2) score < 0.6, Hardy-Weinberg equilibrium $p < 5 \times 10^{-6}$, and mismatch of alleles or allele frequency difference greater than 20% from the Haplotype HRC genome reference panel[29]. Indels and SNPs that were duplicated, multiallelic, monomorphic, or ambiguous (A/T or C/G with MAF > 0.4) were also excluded. Visual inspection of the distribution of the summary statistics was performed, and Manhattan plots and quantile-quantile plots were created for the cleaned summary statistics.

Genomic locus definition. Independently associated loci resulting from the GWAS were further examined using FUMA[36]. Independent significant SNPs, which were identified by a Bonferroni-corrected genome-wide significant two-tailed p value ($p < 5 \times 10^{-8}$), represented

signals that were independent from each other at linkage equilibrium $r^2 < 0.6$. A subset of the independent significant SNPs that showed approximate linkage equilibrium with each other at $r^2 < 0.1$ were defined as 'lead SNPs'. We then defined associated 'genomic loci' by merging any physically overlapping lead SNPs (LD blocks < 250 kb apart). Borders of the associated genomic loci were defined by identifying all SNPs in LD ($r^2 \geq 0.6$) with one of the independent significant SNPs in the locus, and the region containing all of these 'candidate SNPs' was considered to be a single independent genomic locus. LD information was calculated from UKB genotype data [27].

Functional annotation of SNPs. Functional annotation of identified DMN-FC SNPs was examined using FUMA[36]. We selected all candidate SNPs in the associated genomic loci having $r^2 \geq 0.6$ with one of the independent significant SNPs, a suggestive p value ($p < 1 \times 10^{-8}$), and MAF > 0.005 for annotations. Predicted functional consequences for these SNPs were obtained by matching SNPs' chromosome, base-pair position, and reference and alternate alleles to ANNOVAR databases[37], obtaining ANNOVAR categories that identify the SNP's genic position (for example, intron, exon, intergenic) and associated function.

Gene mapping. Genome-wide significant loci obtained by the GWAS analysis were mapped to genes using FUMA[36] according to three strategies: (1) Positional mapping projects SNPs to genes based on physical distance (within a 10-kb window) from known protein-coding genes in the human reference assembly (GRCh37/hg19); (2) eQTL mapping projects SNPs to genes with which they show a significant eQTL association (i.e., allelic variation at the SNP is associated with the expression level of that gene). eQTL mapping uses information from 45 tissue types in 4 data repositories (GTEx[38], Blood eQTL browser[39], BIOS QTL browser[40], psychENCODE[41]) and is based on cis-eQTLs that can map SNPs to genes up to 1 Mb away. We used an FDR of $q < 0.05$ to define significant eQTL associations; (3) Chromatin interaction mapping was performed to map SNPs to genes when there was a 3D DNA-DNA interaction between the SNP region and a gene region. Chromatin interaction mapping can involve long-range interactions, as it does not have a distance boundary. FUMA contained Hi-C data for 14 tissue types from the study of Schmitt et al.[42] and one-way EP data from the psychENCODE[41]. Because chromatin interactions are often defined in a certain resolution, such as 40 kb, an interacting region can span multiple genes. If a SNP is located in a region that interacts with a region containing multiple genes, it will be mapped to each of those genes. To further prioritize candidate genes, we selected only interaction-mapped genes in which one region involved in

the interaction overlapped with a predicted enhancer region in any of the 111 tissue/cell types from the Roadmap Epigenomics project[43] and the other region was located in a gene promoter region (from 250 bp upstream to 500 bp downstream of the TSS and also predicted by Roadmap to be a promoter region). This reduced the number of genes mapped but increased the likelihood that those identified would have a plausible biological function. We used an FDR of $q < 1 \times 10^{-5}$ to define significant interactions, based on previous recommendations[42] modified to account for the differences in cell lines used here.

GWAS catalog lookup. We used FUMA GENE2FUNC[36] to identify SNPs with previously reported ($p < 5 \times 10^{-5}$) phenotypic associations in 56 published GWAS listed in the NHGRI-EBI Catalog[44] that overlapped with the genomic risk loci identified in the current GWAS analysis. The enrichment was tested using a hypergeometric test with a background set of 19,283 genomic protein-coding genes as in FUMA.

Gene-set analysis. MAGMA (v1.07)[45] was used to perform gene-set analysis, which is based on the linear regression model, testing for associations of HAR genes with phenotypes. Conditional gene-set analyses were performed as a follow-up using MAGMA to correct for the effect of BRAIN genes. MAGMA-based gene-set analysis expands hypergeometric enrichment testing in FUMA[36] in the sense that MAGMA weighs the contribution of genes based on the association *p*-value with the trait, whereas in hypergeometric enrichment genes are denoted as ‘implicated’ and then tested for overlap with a gene-set.

Cortical vulnerability in psychiatric disorders

We examined the potential role of evolutionary genes in brain morphology related to psychiatric disorders. A cortical map of disorder involvement was derived from the BrainMap database (http://www.brainmap.org/), describing a large collation of standardized data from voxel-based morphometry (VBM) studies on cortical volume changes in a wide range of brain disorders (994 studies in total)[46, 47, 48]. Five psychiatric disorders were selected (260 studies), including autism spectrum disorder, bipolar disorder, major depressive disorder, obsessive compulsive disorder, and schizophrenia, for which 519, 547, 751, 215, and 2840 disorder-hit voxels (with MNI coordinates) were included. Meta-analyses were conducted for each disorder using the GingerALE toolbox[49, 50]. Resulting brain maps of activation likelihood estimation (ALE) were registered to the MNI 152 template in the FreeSurfer space and regional ALE was

computed by averaging ALEs of all voxels within each cortical region of the DK-114 atlas. Regional averaged ALE was transformed to *Z*-score and then averaged into a cross-disorder cortical involvement map describing per region the level of involvement across the five major psychiatric disorders.

Supplementary Note 1. Alternative identifications of HAR genes

HAR genes identified by brain-related Hi-C and eQTL

The genomic HAR segments as described by Doan et al.[18] were mapped to 184 genes using the eQTL mapping and chromatin interaction mapping defined in brain tissues from the PsychENCODE database[19]. Using these 184 HAR genes, we found that the mean cortical gene expression profile was significantly correlated to the cortical expansion from chimpanzees to humans ($r(53) = 0.321$, $p = 0.017$), but the correlation did not exceed the null distribution of correlation coefficients between gene expression of random ECE genes and cortical expansion ($p = 0.184$, 10,000 permutations). Furthermore, cortical gene expression of regions in higher-order cognitive networks (i.e., DMN, FPN, and VAN) were significantly higher than regions of SMN/VN ($t(44) = 2.883$, $p = 0.010$), with the DMN showing significantly higher gene expression compared to the rest of the cortex ($t(55) = 2.157$, $p = 0.035$). These effects significantly exceeded null distributions of effects obtained by randomly selecting the same sized sets of ECE genes ($p = 0.009$ and 0.019, respectively; 10,000 permutations).

We further examined a subset of genes (32 out of the 184 HAR genes) that overlapped with the 2,979 BRAIN genes. First, cortical gene expression of these 32 HAR-BRAIN genes showed a significant correlation to the cortical expansion from chimpanzees to humans ($r(53) = 0.405$, $p = 0.002$), but this correlation again did not exceed null distributions generated by either random BRAIN genes (NULL1, $p = 0.187$) or random ECE genes (NULL2, $p = 0.052$). Moreover, regions of higher-order cognitive networks showed elevated gene expression as compared to regions of the SMN/VN ($t(44) = 4.026$, $p < 0.001$), with the effect exceeding NULL2 ($p = 0.030$, 10,000 permutations), but not NULL1 ($p = 0.255$, 10,000 permutations). Examining the potential elevation of gene expression in the DMN regions compared to the rest of the cortex also showed a significant effect ($t(55) = 3.240$, $p = 0.002$), exceeding effects from both NULL1 ($p = 0.009$, 10,000 permutations) and NULL2 ($p = 0.003$, 10,000 permutations). These findings suggested that the 32 HAR-BRAIN genes identified according to brain eQTL and Hi-C might play a specific role in differentiating the DMN from other functional networks.

HAR genes identified by MPRA

Using massively parallel reporter assays (MPRA), Doan et al.,[18] identified biallelic HAR mutations related to autism spectrum disorder and mapped these genomic loci to 238 genes according to genomic locations (here further referred to as HAR-ASD genes). In the current study, we used the original list of 238 genes in Doan et al., and did not combine the MPRA results with brain Hi-C/eQTL as it only resulted in 4 genes, which unable to provide enough

statistical power to pick up any effects. First, cortical gene expression profile of these HAR-ASD genes was found to be correlated to cortical expansion from chimpanzees to humans ($r(53) = 0.410$, $p = 0.002$), with the correlation coefficient significantly larger than the null distribution of correlations between cortical expansion and gene expression of random ECE genes ($p = 0.011$, 10,000 permutations). Concerning the seven functional networks, higher-order cognitive networks showed enhanced gene expression of HAR-ASD genes as compared to regions of the SMN/VN ($t(44) = 3.701$, $p < 0.001$), with the DMN regions showing significantly higher gene expression comparing to the rest of the cortex ($t(55) = 2.539$, $p = 0.014$). These effects significantly exceeded effects obtained from random ECE genes (both $p < 0.001$, 10,000 permutations).

Within the 238 HAR-ASD genes, 63 genes were observed to be also described as BRAIN genes (referred to as HAR-ASD-BRAIN genes). The mean cortical gene expression of these 63 genes were significantly correlated to cortical expansion from chimpanzees to humans ($r(53) = 0.484$, $p < 0.001$), exceeding correlations from both NULL1 (i.e., BRAIN genes; $p = 0.021$, 10,000 permutations) and NULL2 (i.e., ECE genes; $p = 0.004$, 10,000 permutations). Furthermore, regions of the higher-order cognitive networks showed an elevated gene expression of HAR-ASD-BRAIN genes as compared to regions of the SMN/VN ($t(44) = 5.871$, $p < 0.001$), an effect significantly larger than effects from NULL1 ($p = 0.031$, 10,000 permutations) and NULL2 ($p = 0.002$, 10,000 permutations). The DMN regions also showed higher gene expression of HAR-ASD-BRAIN genes compared to the rest of the cortex ($t(55) = 3.234$, $p = 0.002$), again, showing a larger effect compared to effects of NULL1 ($p = 0.002$, 10,000 permutations) and NULL2 ($p = 0.004$, 10,000 permutations). These findings suggested that the subset of HAR genes associated with ASD de novo variants might be involved in the process differentiating regions of cognitive systems, in particular the DMN.

Supplementary Note 2. Alternative identifications of DMN genes

In the main text the analysis of the top 200 DMN genes is presented. For validation, the top 53 and 469 genes were examined, showing respectively the highest positive *t* scores (with $p < 0.05$, partial Bonferroni corrected, and $p < 0.01$, not corrected) between the expression level in regions of the DMN and the rest of the cortex. Out of the top 53 and 469 genes (from now on referred to as 53|469), 15|68 were observed in HAR genes (both $p < 0.001$, hypergeometric test), with 6|32 genes found in the subset of HAR-BRAIN genes (both $p < 0.001$, hypergeometric test). Permutation analysis by shuffling region labels across the seven functional networks revealed a significant enrichment of HAR genes in the top DMN genes (*p*

< 0.001, for both top 53 and 469 DMN genes; 10,000 permutations). Using the the hypergeometric test[36], we again observed neuron-related GO annotations for the top 53|469 DMN genes, with results tabulated in Supplementary Table 12 and 13.

We further examined to what extent the top 53|469 DMN genes were associated with the genetic variants of intelligence, social behaviour, and schizophrenia. As a result, the top 53 DMN genes were found to be significantly associated with genetic variants of Frequency of friend/family visits (β = 0.013, p = 0.022) and schizophrenia (β = 0.017, p = 0.014); no clear significant effect for intelligence could be observed (β = -0.001, p = 0.568). The top 469 DMN genes showed significant associations with all three phenotypes (intelligence: β = 0.022, p = 0.004; Frequency of friend/family visits: β = 0.015, p = 0.008; schizophrenia: β = 0.015, p = 0.026).

Supplementary Note 3. Examination of the potential effects of tissue sample number

In the AHBA dataset used in the principal analysis, a distinct number of tissue samples was obtained within each of the 114 DK regions (mean [SD] = 3.3 [2.5], ranging from 1 ~ 13). Gene expression profiles of tissue samples were averaged within brain regions across different numbers of tissue samples. Here we verified our key results of HAR-BRAIN gene expression considering the potential effects of variations in the number of tissue samples in each cortical region.

First, we regressed out the number of tissue samples from the gene expression profile using the linear regression model and found similar results using the residuals of gene expression. We found that the HAR-BRAIN gene expression was significantly correlated with cortical expansion (r(55) = 0.491 , p < 0.001; p < 0.001 for both NULL1 and NULL2, 10,000 permutations). Moreover, regions in higher-order cognitive networks (i.e., DMN, FPN, and VAN combined) showed higher HAR-BRAIN gene expression as compared to the SMN/VN (t(44) = 4.657, p < 0.001; p = 0.013 and p < 0.001 for NULL1 and NULL2, respectively, 10,000 permutations), with the DMN regions showing the highest gene expression compared to the rest of the cortex (t(55) = 3.154, p = 0.003; p < 0.001 for both NULL1 and NULL2, 10,000 permutations).

Second, we performed 1,000 randomizations, in which a single tissue sample was randomly selected per region, and the gene expression profile of the selected sample was used to represent the profile of the corresponding DK region. Across 1,000 randomizations, we observed an averaged t(44) = 4.401 (p < 0.001) when comparing gene expressions of the 415 HAR-BRAIN genes between regions in higher-order cognitive networks and SMN/VN, as well

as an averaged $t(55) = 2.436$ ($p = 0.018$) between regions of the DMN and the rest of the cortex. Furthermore, the pattern of HAR-BRAIN gene expression was correlated to the pattern of chimpanzee-to-human cortical expansion, with an averaged correlation coefficient of $r = 0.369$ ($p = 0.005$). These findings confirmed the high-level HAR-BRAIN gene expression in cognitive functional networks and its association with evolutionary cortical expansion.

Supplementary Note 4. Alternative cortical parcellation atlases

BB-38 atlas for chimpanzee-human comparison

We validated the association between cortical evolutionary expansion and HAR gene expression using the BB-38 atlas that describes homologous cortical areas across the two species. Cortical expansion of the seven resting-state functional networks are displayed in Supplementary Figure 14a, showing the largest expansion in the FPN and the second largest in the DMN. Regions of the higher-order cognitive networks demonstrated larger expansion compared to the SMN/VN ($t(28) = 3.632$, $p < 0.001$; Supplementary Figure 14b). Furthermore, we found higher gene expression levels for the set of HAR-BRAIN genes in regions of cognitive networks comparing to the SMN/VN ($t(28) = 2.572$; $p = 0.016$; Supplementary Figure 14c). Cortical gene expression of HAR-BRAIN genes was significantly correlated with cortical evolutionary expansion ($r(28) = 0.400$, $p = 0.014$, Supplementary Figure 14d), suggesting consistent findings between the BB-38 atlas and DK atlas.

DK-219 atlas

Results were further validated using a finer subdivision of DK atlas consisting of 219 cortical regions (111 regions in left hemisphere)[6, 7]. Regions of the DMN, FPN, and VAN consistently showed larger cortical surface area expansion as compared to regions of the SMN/VN ($t(171) = 3.671$, $p < 0.001$). A similar effect was also observed when we compared regions of the DMN with the SMN/VN ($t(124) = 2.895$, $p = 0.005$). The pattern of chimpanzee-to-human cortical expansion consistently correlated with the pattern of gene expression of HAR-BRAIN genes ($r(109) = 0.390$, $p < 0.001$), again, significantly exceeding null conditions in which similar sized set of BRAIN genes were randomly selected ($p < 0.001$ for HAR-BRAIN genes; 10,000 permutations). Significantly enhanced HAR-BRAIN gene expression remained in cognitive network regions as compared to the SMN/VN regions ($t(84) = 5.293$, $p < 0.001$), as well in the DMN regions compared to the rest of the cortex ($t(109) = 2.247$, $p = 0.027$), with effects remaining significant in permutation testing conditional to BRAIN genes ($p = 0.016$ and $p < 0.001$, separately, 10,000 permutations).

Supplementary Note 5. Alternative functional network divisions

Yeo-2011 17 functional networks

We validated our results using a finer functional network division that consists of 17 functional networks[20]. Four out of 17 functional networks (labeled as 11, 15, 16, and 17 in [20]) were identified as components of the DMN, as shown in Supplementary Figure 11. A group of these DMN components and networks labeled as 7, 8, 12, and 13 was defined as higher-order cognitive networks, and networks labeled as 1, 2, 3, and 4 were defined as SMN/VN. Using this functional network division, a significantly larger cortical expansion in regions of higher-order cognitive networks as compared to SMN/VN was validated ($t(82) = 2.185$, $p = 0.032$). Regions in cognitive networks consistently exhibited higher gene expression for HAR-BRAIN genes ($t(40) = 5.182$, $p < 0.001$), as compared to regions of the SMN/VN. Comparing the DMN regions to the rest of the cortex showed similar results ($t(55) = 2.306$, $p = 0.025$). These effects remained significant in permutation testing conditional to BRAIN genes ($p = 0.009$ and $p = 0.003$, separately, 10,000 permutations).

Raichle's default-mode network

We additionally validated our findings by using a refined DMN division according to Raichle[21]. We manually identified 14 cortical regions in DK atlas as default-mode network regions, including regions located in precuneus, middle temporal lobe, inferior parietal lobe, and medial frontal lobe (Supplementary Figure 11). Under this division, we consistently observed significantly enhanced HAR-BRAIN gene expression in the DMN regions as compared to the rest of the cortex ($t(55) = 2.476$, $p = 0.016$). Permutation analysis by randomly selecting the similar sized set of BRAIN genes or ECE genes also showed consistent results ($p = 0.003$ and $p < 0.001$, 10,000 permutations; Supplementary Figure 10).

Supplementary Note 6. Directly mapping AHBA tissue samples to functional networks

We validated our findings by directly mapping the ~400 tissue samples of each donor in AHBA dataset to the functional networks, without the intermediate step of mapping both sets to the DK-114 atlas. Gene expression profiles of tissue samples within the same functional network were averaged, resulting in a 7 × 20,734 matrix representing the mean gene expression for each gene per functional network. A 7 × 1,711 sub-matrix of gene expressions of HAR genes was further selected. We then examined whether HAR genes showed the highest expression level in the default-mode network by performing two-sided paired-sample *t* tests across HAR genes

between the DMN and each of 6 other functional networks. This analysis showed the highest HAR gene expressions in the DMN, as compared with the visual ($t(1,710) = 2.517$, $p = 0.012$), somatomotor ($t(1,710) = 10.369$, $p < 0.001$), dorsal-attention ($t(1,710) = 2.294$, $p = 0.021$), ventral-attention ($t(1,710) = 11.267$, $p < 0.001$), limbic ($t(1,710) = 2.392$, $p = 0.017$, and frontoparietal networks ($t(1,710) = 7.844$, $p < 0.001$). Similar findings were observed when we considered the subset of 415 HAR-BRAIN genes ($t(414) = 3.682$, $p < 0.001$, visual; $t(414) = 10.323$, $p < 0.001$, somatomotor; $t(414) = 2.394$, $p = 0.017$, dorsal-attention; $t(414) = 7.463$, $p < 0.001$, ventral-attention; $t(414) = 5.873$, $p < 0.001$, frontoparietal, except for the limbic network, $t(414) = -0.419$, $p = 0.676$).

Given the 7 × 415 (function networks × HAR-BRAIN genes) expression matrix, we next averaged gene expression levels across the default-mode, frontoparietal, and ventral-attention networks, resulting in a 1 × 415 vector of expression levels of HAR-BRAIN genes in higher-order cognitive functional networks. Similarly, gene expressions were averaged across primary visual and somatomotor networks. Paired-sample *t*-test was performed between the two resulting gene expression profiles and showed that HAR-BRAIN gene expression was significantly higher in cognitive networks as compared with regions of the SMN/VN ($t(414) = 5.345$, $p < 0.001$). Also, comparing the gene expression profile of the DMN with the mean profile of the other 6 functional networks showed significantly higher HAR-BRAIN gene expression in the DMN ($t(414) = 8.922$, $p < 0.001$). We further performed a permutation test by selecting similar sized set of random BRAIN genes from the original 7 × 20,734 expression matrix and recomputing the paired-sample *t* test between the mean expression profiles of higher-order cognitive and primary networks, as well as between the default-mode network and others, for 10,000 times. The original *t* scores significantly exceeded 10,000 permutations for both conditions ($p = 0.003$ for the comparison between cognitive and primary networks; $p < 0.001$ for the comparison between the DMN and others). Taking together, this validation analysis confirmed that cognitive functional networks, in particular the DMN showed the highest level of HAR/HAR-BRAIN gene expression.

Supplementary Note 7. Alternative parameters in processing

Distance threshold for tissue sample inclusion

In the main analysis, a distance threshold of 2 mm was used to map tissue samples in the AHBA data to the gray matter. We alternatively used thresholds of 3 mm and 1 mm to validate our findings. For both thresholds, regions in higher-order DMN, FPN, and VAN consistently showed higher HAR-BRAIN gene expression as compared to the SMN/VN regions ($t(44) =$

3.911, $p < 0.001$, and $t(44) = 5.522$, $p < 0.001$, for 1mm and 3mm, respectively). Comparing the DMN regions to the rest of the cortex showed similar results ($t(44) = 2.897$, $p = 0.054$, and $t(55) = 2.790$, $p = 0.072$, for 1mm and 3mm, respectively). Permutation analyses by selecting similar sized sets of random BRAIN genes consistently showed significance ($p = 0.014$ and 0.009, for threshold 1mm and 3 mm, separately, in the comparison between cognitive and the SMN/VN regions; $p < 0.001$ for both thresholds in the comparison between default-mode network regions and others; 10,000 permutations). Furthermore, the cortical expression pattern of HAR-BRAIN genes was also significantly correlated with chimpanzee-to-human cortical expansion ($r(55) = 0.334$ and 0.363, $p = 0.011$ and 0.006, for 1mm and 3 mm, respectively), with consistent results observed when performing permutation testing ($p < 0.001$ for both thresholds, 10,000 permutations). In addition, the cortical expression pattern of HAR-BRAIN genes were consistently associated with the pattern of cortical vulnerability to psychiatric disorders for both settings ($r(55) = 0.300$, $p = 0.026$, and $r(55) = 0.319$, $p = 0.018$, for 1 mm and 3mm, respectively). Effects were significantly larger than seen in the null condition of randomly selected BRAIN genes ($p = 0.006$ and $p = 0.030$, for 1mm and 3 mm, respectively; 10,000 permutations).

FDR correction in BRAIN genes selection

In addition to setting FDR $q < 0.05$ for selecting GTEx BRAIN genes in the main text, we alternatively applied FDR $q < 0.01$ and $q < 0.001$ to identify BRAIN genes from the GTEx dataset. At these two thresholds, respectively 2,544 and 2,102 genes were identified as BRAIN genes, from which 368 and 305 genes could be denoted as HAR-BRAIN genes. For both thresholds, higher-order cognitive network regions remained to show higher HAR-BRAIN gene expression as compared to the SMN/VN regions ($t(44) = 5.155$, $p < 0.001$ and $t(44) = 5.317$, $p < 0.001$, for FDR $q < 0.01$ and $q < 0.001$, respectively), with similar effects found when we compared regions of the DMN to the rest of the cortex ($t(55) = 3.269$, $p = 0.002$ and $t(55) = 3.330$, $p = 0.002$, for FDR $q < 0.01$ and $q < 0.001$, respectively). These effects remained significant in permutation testing in which random BRAIN genes were selected ($p = 0.008$ and 0.013, for FDR $q < 0.01$ and 0.001, respectively, in the comparison between cognitive and primary networks; $p < 0.001$, for both thresholds, in the comparison between the DMN and others). Correlating the pattern of HAR-BRAIN gene expression to the pattern of cortical expansion consistently showed significant correlations ($r(55) = 0.392$, $p = 0.003$ and $r(55) = 0.382$, $p = 0.003$, for FDR q < 0.01 and 0.001, respectively), with both effects significant in permutation testing conditional to BRAIN genes ($p = 0.001$ for both thresholds, 10,000

permutations). Furthermore, cortical HAR-BRAIN gene expression was significantly associated with the cortical pattern of disorder vulnerability ($r(55) = 0.354$, $p = 0.008$ and $r(55) = 0.327$, $p = 0.015$ for FDR $q < 0.01$ and 0.001, respectively), effects again exceeding effects of randomly selected BRAIN genes ($p = 0.016$ and 0.049 for $q < 0.01$ and 0.001, respectively).

Supplementary Note 8. HAR-BRAIN gene expression in subcortical regions

In the main text, we demonstrated differentiated gene expression of HAR-BRAIN genes in cortical regions of higher-order cognitive networks. Here we additionally examined whether HAR-BRAIN genes showed differentiated gene expression in cortical regions as compared to subcortical regions. To this end, we mapped subcortical AHBA tissue samples to seven subcortical regions delineated in the DK atlas, including thalamus, caudate, putamen, pallidum, hippocampus, amygdala, and nucleus accumbens. We noticed that subcortical regions showed a significantly lower HAR-BRAIN gene expression as compared to cortical regions ($t(62) = 3.427$, $p = 0.001$). This effect exceeded null distribution of effects obtained by random ECE genes (NULL1, $p < 0.001$, 10,000 permutations), but did not exceed null distribution of effects obtained by random BRAIN genes (NULL2, $p = 0.712$, 10,000 permutations). This suggested that HAR-BRAIN genes contributed to differentiating cortical regions from subcortical regions to a similar extent as genes generally involved in brain processes.

Supplementary Note 9. Discussion on evolutionarily cortical expansion

Our results showed high levels of cortical expansion in regions of both the FPN and DMN in humans. To identify cortical regions with significantly large expansion, permutation testing was performed by comparing the observed cortical expansion from chimpanzees to humans to null distributions of expansions computed by randomly shuffling the brain regions in the two species. This analysis showed significantly larger expansion in bilateral rostral middle frontal lobe, orbital inferior frontal gyrus, and right inferior/superior parietal lobe, anterior cingulate gyrus, and triangular part of inferior frontal gyrus (all uncorrected $p < 0.001$; FDR correction, $q < 0.05$; 10,000 permutations; Supplementary Figure 13), which are compatible with prior observations of cortical variation between macaque and human[51, 52]. The discrepancy between our findings and prior studies[51, 52] was a relatively low expansion found in the middle/posterior cingulate cortex (×1.5 expansion, adjusted $p < 0.001$), potentially attributable to the involvement of cingulate cortex in the paleomammalian brain that arose early in mammalian evolution[53]. Our chimpanzee-human comparison also demonstrated a relatively large expansion of the lingual gyrus in humans, which might attribute to humans' largely evolved

functions of word processing and language compared to chimpanzees[54]. Furthermore, our study used MRI data of 29 adult chimpanzees with a mean (SD) age of 30.2 (12.6) and 30 adult humans with a mean (SD) age of 30.2 (3.1). We examined the potential age and sex effect on our main cortical expansion results as follows.

First, we normalized the age within each species, separately, by dividing the age of humans by 80 (an approximately mean age of human lifespan) and dividing the age of chimpanzees by 40 (an approximately mean age of captive chimpanzees). Then we regressed out the age from the surface area of each region using the linear regression model and computed cortical expansion between the two species. We found that cortical expansion of regions of higher-order cognitive networks remained to be significantly higher than the SMN/VN regions ($t(86) = 2.667$, $p = 0.009$). The FPN still showed the highest expansion (FPN versus the rest of the cortex: $t(108) = 3.092$, $p = 0.003$; FDR corrected), with the DMN ranked in the second place (DMN versus the rest of the cortex: $t(108) = 2.105$, $p = 0.038$; not corrected). Moreover, the cortical expansion significantly correlated with HAR-BRAIN gene expression ($r(53) = 0.440$, $p < 0.001$), with the correlation coefficient exceeding both NULL1 ($p = 0.012$) and NULL2 ($p = 0.002$). These findings suggest that the age effect did not drive our cortical expansion results.

We exclusively included MRI data of female chimpanzee and female human subjects, due to the practical reason for greater availability of female chimpanzees. Female human subjects were randomly selected from the HCP database to match the chimpanzee sample. Although it was not within the scope of the current study, it is possible that there are potential sex effects in the pattern of cortical expansion between humans and chimpanzees. Future work including sufficiently large chimpanzee samples of both male and female subjects may help to elucidate potential sex differences in cross-species cortical expansion.

Supplementary Tables

Supplementary Table 1. Cortical expansion in each functional network

functional network	normalized cortical expansion		*t* score	effect size (cohen's *d*)	adjusted *p*-value
	mean	std			
VN	0.0007	0.2192	-0.6858	-0.1724	0.6919
SMN	-0.1	0.15	-2.9843	-0.75	**0.0122**
DAN	0.0219	0.1803	-0.1469	-0.051	0.8835
VAN	-0.0244	0.1374	-1.1175	-0.3013	0.4658
LN	0.0209	0.2306	-0.215	-0.0596	0.8835
FPN	0.2938	0.2104	3.4144	1.3321	**0.0062**
DMN	0.1167	0.2314	2.4604	0.5291	**0.0359**

BOLD: significant

Supplementary Table 2. HAR gene expression in each functional network

functional network	gene expression		*t* score	effect size (cohen's *d*)	adjusted *p*-value
	mean	std			
VN	0.0027	0.0621	0.2403	0.0916	0.811
SMN	-0.0765	0.0775	-3.7018	-1.2891	**0.004**
DAN	-0.0174	0.0925	-0.38	-0.197	0.811
VAN	0.0051	0.0737	0.3114	0.1257	0.811
LN	0.0165	0.072	0.733	0.2958	0.811
FPN	-0.0186	0.0609	-0.3532	-0.2095	0.811
DMN	0.0292	0.0639	2.2737	0.6479	0.0718

BOLD: significant

Supplementary Table 3. HAR-BRAIN gene expression in each functional network

functional network	gene expression		*t* score	effect size (cohen's *d*)	adjusted *p*-value
	mean	std			
VN	-0.0600	0.1145	-1.0632	-0.4054	0.4385
SMN	-0.1843	0.1257	-4.9206	-1.7136	**8.21E-06**
DAN	-0.0115	0.1654	-0.0417	-0.0216	0.9669
VAN	0.0024	0.1371	0.2064	0.0833	0.9419
LN	0.0596	0.1551	1.3078	0.5278	0.3535
FPN	0.0123	0.0584	0.2483	0.1473	0.9419
DMN	0.0785	0.0923	3.2673	0.9310	**0.0042**

BOLD: significant

Supplementary Table 4. Over-representation of the top 200 DMN genes

Gene ontology term	*N*	*n*	adjusted *p*-value	gene symbol
Cellular Components				
Dendrite	451	14	2.60E-05	*FAS, NELL2, ITPKA, KCNJ12, SLC8A2, CHL1, GLRA3, KCNN2, PNOC, KCNB2, CALB1, NOV, NTRK2, SLC9A6*
Somatodendritic Compartment	649	17	4.49E-05	*KNCN, FAS, NELL2, ITPKA, KCNJ12, SLC8A2, CHL1, GLRA3, KCNN2, VIP, PNOC, CRH, KCNB2, CALB1, NOV, NTRK2, SLC9A6*
Cell Body	493	14	7.15E-05	*KNCN, FAS, NELL2, KCNJ12, SLC8A2, GLRA3, KCNN2, VIP, PNOC, CRH, KCNB2, CALB1, NOV, NTRK2*
Perikaryon	108	6	7.45E-05	*NELL2, SLC8A2, GLRA3, CRH, KCNB2, NTRK2*
Rnai Effector Complex	11	2	1.29E-04	*DCP2, SND1*
Synapse Part	607	15	2.15E-04	*SVOP, ITPKA, BAIAP3, NETO2, CBLN1, CDH8, ADCYAP1, SLC8A2, SEMA4F, LRRTM4, GLRA3, KCNN2, STX1A, CALB1, NTRK2*
Neuron Projection Terminus	129	6	2.26E-04	*CDH8, ADCYAP1, PNOC, CALB1, NTRK2, SLC9A6*
Synapse	751	17	2.78E-04	*OLFM3, SVOP, ITPKA, BAIAP3, NETO2, CBLN1, CDH8, ADCYAP1, SLC8A2, SEMA4F, LRRTM4, GLRA3, KCNN2, STX1A, CALB1, NTRK2, SLC9A6*
Cullin Ring Ubiquitin Ligase Complex	148	6	5.20E-04	*FBXO6, KLHL12, CDC16, DCAF11, DCAF4, FBXL2*
T Tubule	45	3	8.47E-04	*CACNB2, KCNJ12, KCNN2*
Molecular Functions				
Neuropeptide Hormone Activity	28	4	5.85E-06	*ADCYAP1, VIP, PNOC, CRH*
Calcium Activated Potassium Channel Activity	17	3	1.66E-05	*KCNT2, PKD2L1, KCNN2*
Calcium Activated Cation Channel Activity	28	3	1.32E-04	*KCNT2, PKD2L1, KCNN2*

N: Gene set size; *n*: number of contained candidates

Supplementary Table 5. Genes annotated from the GWAS on DMN functional activity

gene symbols	chr	start	end	min GWAS *p*-value
FFAR4	10	95326422	95364237	9.25E-14
LGI1	10	95517566	95557916	9.25E-14
SLC35G1	10	95653730	95715819	9.25E-14
PLCE1	10	95753746	96092580	9.25E-14
NOC3L	10	96075004	96122716	9.25E-14
TBC1D12	10	96162261	96295687	9.25E-14
HELLS	10	96305547	96373662	9.25E-14
DPYSL4	10	134000404	134019280	8.29E-10
STK32C	10	134020996	134145351	2.61E-10
LRRC27	10	134145614	134195010	8.27E-10
PWWP2B	10	134210672	134231367	2.61E-10
INPP5A	10	134351324	134596979	2.61E-10

Supplementary Table 6. Over-representation of DMN functional activity associated genes in GWAS catalog reported gene-sets

gene set	*N*	*n*	adjusted *p*-value	gene symbols
Plasma clozapine-norclozapine ratio in treatment-resistant schizophrenia	16	5	2.63E-07	PLCE1, NOC3L, TBC1D12, HELLS
Thiopurine-induced alopecia in inflammatory bowel disease	11	2	1.64E-02	TBC1D12, HELLS

N: Gene set size; *n*: number of contained candidates

Supplementary Table 7. Enrichment of HAR, HAR-BRAIN, BRAIN, and DMN genes in GWAS results

Gene set	*N*	*b*	*β*	SE	*p*-value	adjusted *p*-value
DMN functional activity						
HAR	1522	0.039	0.011	0.024	5.04E-02	8.19E-02
HAR-BRAIN	379	0.103	0.015	0.048	1.60E-02	**3.78E-02**
BRAIN	2638	0.012	0.004	0.016	2.26E-01	2.56E-01
DMN (200)	177	0.061	0.006	0.060	1.56E-01	1.84E-01
DMN-HAR-BRAIN (37)	37	-0.040	-0.002	0.145	6.08E-01	6.32E-01
Intelligence						
HAR	1509	0.218	0.058	0.036	5.43E-10	**3.53E-09**
HAR-BRAIN	377	0.546	0.075	0.069	1.22E-15	**3.18E-14**
BRAIN	2624	0.177	0.060	0.024	2.35E-13	**3.06E-12**
DMN (200)	176	0.133	0.012	0.086	6.15E-02	9.41E-02
DMN-HAR-BRAIN (37)	36	0.514	0.022	0.216	8.81E-03	**2.55E-02**
Frequency of friend/family visits						
HAR	1508	0.139	0.037	0.027	1.2E-07	**5.36E-07**
HAR-BRAIN	377	0.28	0.039	0.054	1.0E-07	**5.25E-07**
BRAIN	2623	0.052	0.018	0.018	1.7E-03	**5.61E-03**
DMN (200)	176	0.126	0.012	0.063	2.4E-02	**4.70E-02**
DMN-HAR-BRAIN (37)	36	0.32	0.014	0.159	2.2E-02	**4.70E-02**
Schizophrenia						
HAR	1508	0.072	0.019	0.032	1.33E-02	**3.46E-02**
HAR-BRAIN	376	0.308	0.043	0.063	5.06E-07	**1.88E-06**
BRAIN	2622	0.146	0.050	0.022	1.69E-11	**1.46E-10**
DMN (200)	176	0.115	0.011	0.077	6.66E-02	9.61E-02
DMN-HAR-BRAIN (37)	36	0.127	0.005	0.199	2.61E-01	2.83E-01

N: Number of genes; *b*: regression coefficient; *β*: standardized regression coefficient; SE: standard error; *p* values adjusted for FDR. BOLD: significant.

Supplementary Table 8. Enrichment of HAR-BRAIN genes in GWAS results of "NETMAT amplitude"

phenotype	functional network	*b*	*β*	SE	*p*-value	adjusted *p*-value
NETMAT amplitude 25(01)	DMN	0.1027	0.0147	0.0479	0.0160	**0.0378**
NETMAT amplitude 25(02)	VN	0.0916	0.0131	0.0474	0.0267	0.0819
NETMAT amplitude 25(03)	VAN	0.0495	0.0071	0.0478	0.1504	0.1844
NETMAT amplitude 25(05)	FPN.R	-0.0378	-0.0054	0.0476	0.7869	0.7869
NETMAT amplitude 25(06)	FPN.L	0.0611	0.0087	0.0477	0.1000	0.1369
NETMAT amplitude 25(10)	SMN	0.0549	0.0079	0.0474	0.1233	0.1602
NETMAT amplitude 25(14)	LN	0.0784	0.0112	0.0477	0.0502	0.0819

N: Number of genes; *b*: regression coefficient; *β*: standardized regression coefficient; SE: standard error; *p* values adjusted for FDR. BOLD: significant.

Supplementary Table 9. Over-representation of HAR genes

Gene ontology term	N	n	adjusted *p*-value
Neuron Part	1261	525	1.73E-138
Synapse	751	378	2.49E-130
Synapse Part	607	325	1.52E-121
Neuron Projection	940	406	1.02E-111
Postsynapse	376	202	1.50E-75
Cell Projection	1778	528	1.35E-72
Somatodendritic Compartment	649	274	7.64E-72
Axon	417	210	9.12E-72
Synaptic Membrane	259	158	3.72E-70
Presynapse	282	157	7.77E-62
Dendrite	451	203	7.81E-59
Cell Projection Part	944	320	6.80E-57
Postsynaptic Membrane	203	124	1.71E-55
Axon Part	218	128	2.24E-54
Excitatory Synapse	196	117	6.16E-51
Cell Body	493	195	5.88E-46
Transporter Complex	318	147	1.17E-44
Plasma Membrane Region	926	272	1.91E-35
Membrane Region	1129	310	3.17E-34
Cell Junction	1146	311	2.65E-33

N: Gene set size; *n*: number of contained candidates; Note: only top 20 cellular components tabulated

Supplementary Table 10. Demographics of human donors included in the AHBA

donor ID	number of samples	sex	age (years)	race/ethnicity
H0351.2001	946	Male	24	African American
H0351.2002	893	Male	39	African American
H0351.1009	363	Male	57	Caucasian
H0351.1012	529	Male	31	Caucasian
H0351.1015	470	Female	49	Hispanic
H0351.1016	501	Male	55	Caucasian

Supplementary Table 11. Demographics of human, chimpanzee, and macaque specimens included in the PsychENCODE dataset

number	species	sex	age	stage	hemisphere
HSB123	Homo sapiens	Male	37	Adulthood	Right
HSB126	Homo sapiens	Female	30	Adulthood	Right
HSB130	Homo sapiens	Female	21	Adulthood	Left
HSB145	Homo sapiens	Male	36	Adulthood	Right
HSB135	Homo sapiens	Female	40	Adulthood	Right
HSB136	Homo sapiens	Male	23	Adulthood	Right
PTB162	Pan troglodytes	Female	22.5	Adulthood	Left
PTB164	Pan troglodytes	Female	30.8	Adulthood	Right
PTB165	Pan troglodytes	Male	31.2	Adulthood	Right
PTB166	Pan troglodytes	Male	26.4	Adulthood	Right
PTB167	Pan troglodytes	Male	29.8	Adulthood	Right
RMB160	Macaca mulatta	Female	10.7	Adulthood	Left
RMB161	Macaca mulatta	Male	11	Adulthood	Left
RMB196	Macaca mulatta	Female	11	Adulthood	Right
RMB218	Macaca mulatta	Male	7	Adulthood	Left
RMB219	Macaca mulatta	Male	7	Adulthood	Left

Supplementary Table 12. Over-representation of the top 53 DMN genes

gene ontology term	*N*	*n*	adjusted *p*-value
Biological Processes			
Negative Regulation of Leukocyte Chemotaxis	13	2	3.12E-06
Regulation of Monocyte Chemotaxis	20	2	1.23E-05
Regulation of System Process	506	7	1.65E-05
Adherens Junction Organization	70	3	1.94E-05
Adenylate Cyclase Activating G Protein Coupled Receptor Signaling Pathway	72	3	2.17E-05
Positive Regulation of Camp Metabolic Process	89	3	5.00E-05
Negative Regulation of Leukocyte Migration	32	2	5.25E-05
Negative Regulation of Potassium Ion Transport	32	2	5.25E-05
Positive Regulation of Vasodilation	32	2	5.25E-05
Regulation of Sensory Perception	36	2	7.52E-05
Negative Regulation of Inflammatory Response	99	3	7.59E-05
Negative Regulation of Transcription Factor Import Into Nucleus	39	2	9.57E-05
Positive Regulation of Cyclic Nucleotide Metabolic Process	109	3	1.10E-04
Positive Regulation of Adenylate Cyclase Activity	48	2	1.79E-04
Regulation of Vasodilation	48	2	1.79E-04
Negative Regulation of Chemotaxis	50	2	2.02E-04
Regulation of Camp Metabolic Process	129	3	2.11E-04
Positive Regulation of Nucleotide Metabolic Process	132	3	2.31E-04
Regulation of Myotube Differentiation	55	2	2.68E-04
Neuron Neuron Synaptic Transmission	56	2	2.83E-04
Negative Regulation of Defense Response	143	3	3.13E-04
Adenylate Cyclase Modulating G Protein Coupled Receptor Signaling Pathway	144	3	3.21E-04
Positive Regulation of Lyase Activity	61	2	3.64E-04
Homophilic Cell Adhesion Via Plasma Membrane Adhesion Molecules	149	3	3.66E-04
Negative Regulation of Response To External Stimulus	272	4	3.73E-04
Cell Cell Adhesion	600	6	3.85E-04
Negative Regulation of Response To Wounding	155	3	4.25E-04
Regulation of Cyclic Nucleotide Metabolic Process	155	3	4.25E-04
Regulation of Neurological System Process	68	2	5.01E-04
Regulation of Blood Circulation	295	4	5.40E-04
Regulation of Adenylate Cyclase Activity	70	2	5.46E-04
Single Organism Cell Adhesion	455	5	5.52E-04
Negative Regulation of Transport	455	5	5.52E-04
Negative Regulation of Nucleocytoplasmic Transport	71	2	5.69E-04
Smoothened Signaling Pathway	72	2	5.93E-04
G Protein Coupled Receptor Signaling Pathway Coupled to Cyclic Nucleotide Second Messenger	171	3	6.15E-04
Actin Mediated Cell Contraction	73	2	6.17E-04
Negative Regulation of Hormone Secretion	74	2	6.42E-04
Cell Junction Organization	183	3	7.93E-04

Cardiac Conduction	82	2	8.66E-04
Regulation of Potassium Ion Transport	83	2	8.97E-04
Cellular Components			
Transporter Complex	318	5	8.00E-05
T Tubule	45	2	1.47E-04
Ionotropic Glutamate Receptor Complex	47	2	1.68E-04
Calcium Channel Complex	62	2	3.82E-04
Molecular functions			
Neuropeptide Hormone Activity	28	2	3.49E-05

N: Gene set size; *n*: number of contained candidates

Supplementary Table 13. Over-representation of the top 469 DMN genes

gene ontology term	*N*	*n*	adjusted *p*-value
Cellular Components			
Synapse Part	607	31	2.93E-06
Perikaryon	108	11	3.48E-06
Somatodendritic Compartment	649	32	4.42E-06
Dendrite	451	25	5.32E-06
Neuron Projection	940	40	1.32E-05
Synapse	751	34	1.49E-05
Axon Part	218	15	2.02E-05
Neuron Projection Terminus	129	11	2.18E-05
Postsynapse	376	21	2.25E-05
Terminal Bouton	64	7	6.43E-05
Cell Body	493	24	6.78E-05
Neuron Part	1261	47	7.80E-05
Axon	417	21	1.05E-04
Neuron Spine	121	9	2.79E-04
Cullin Ring Ubiquitin Ligase Complex	148	10	3.54E-04
Postsynaptic Membrane	203	12	4.55E-04
Ubiquitin Ligase Complex	260	14	5.18E-04
Cell Projection Part	944	35	6.12E-04
Myosin Filament	22	3	1.12E-03
Nuclear Replication Fork	39	4	1.39E-03
Rnai Effector Complex	11	2	1.42E-03
Filopodium Tip	11	2	1.42E-03
Cul4 Ring E3 Ubiquitin Ligase Complex	25	3	1.84E-03
Molecular functions			
Ubiquitin Like Protein Ligase Activity	197	14	2.38E-05

N: Gene set size; *n*: number of contained candidates

Supplementary Figures

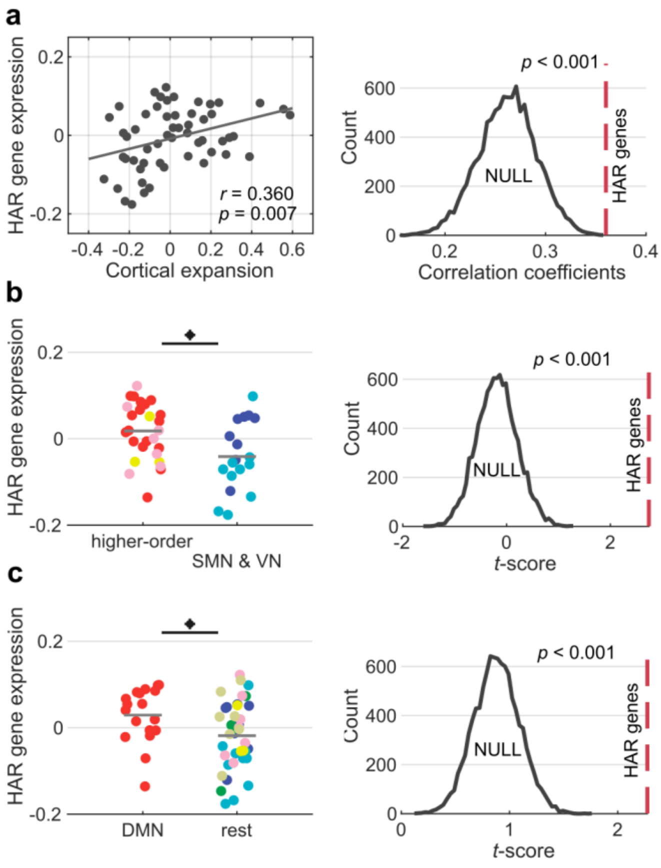

Supplementary Figure 1. *HAR gene expression*. (a) Cortical HAR gene expression is significantly correlated with the normalized chimpanzee-human cortical expansion ($r(53) = 0.360$, $p = 0.007$) (left), with the effect significantly exceeding the null condition generated by ECE genes ($p < 0.001$, 10,000 permutations) (right). (b) Cortical HAR gene expression in regions of higher-order networks is significantly higher than regions of the SMN/VN ($t(55) = 2.742$, $p = 0.009$) (left), with the effect above the null condition generated by ECE genes ($p < 0.001$, 10,000 permutations) (right). (c) Cortical HAR gene expression in regions of DMN is marginally higher than the rest of the cortex ($t(55) = 2.274$, $p = 0.027$, uncorrected) (left), also, with the effect significantly exceeding the null condition generated by ECE genes ($p < 0.001$, 10,000 permutations) (right). Colors indicate functional networks, as in Fig. 2 in the main text. Source data are provided as a Source Data file.

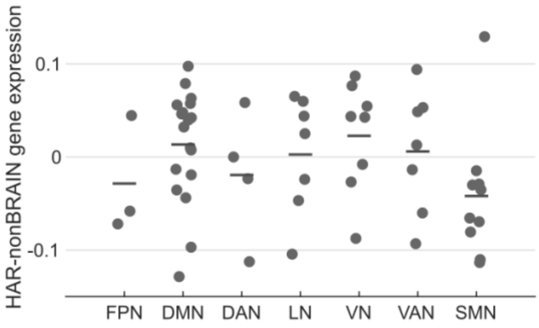

Supplementary Figure 2. *HAR-nonBRAIN gene expression*. HAR-nonBRAIN gene expression within each of the seven functional networks, ranked in descending order according to their mean expansion per network (as in Fig. 2c). Source data are provided as a Source Data file.

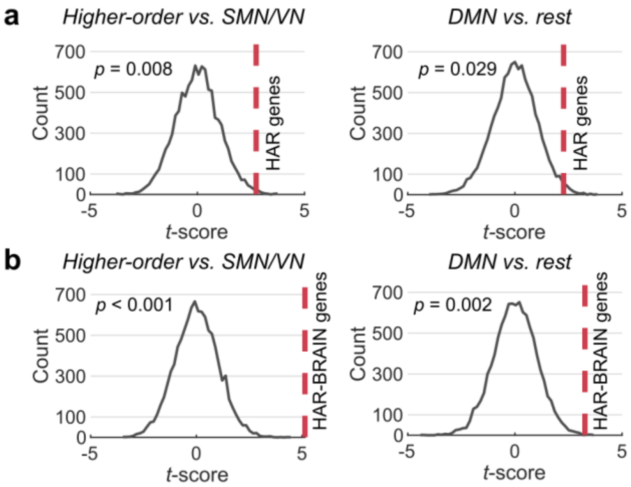

Supplementary Figure 3. *Permutation testing of HAR/HAR-BRAIN gene expression by randomly shuffling cortical regions*. (a) HAR gene expression between higher-order networks and the SMN/VN (left panel) and between the DMN and the rest of the cortex (right panel). (b) HAR-BRAIN gene expression between higher-order networks and the SMN/VN (left panel) and between the DMN and the rest of the cortex (right panel).

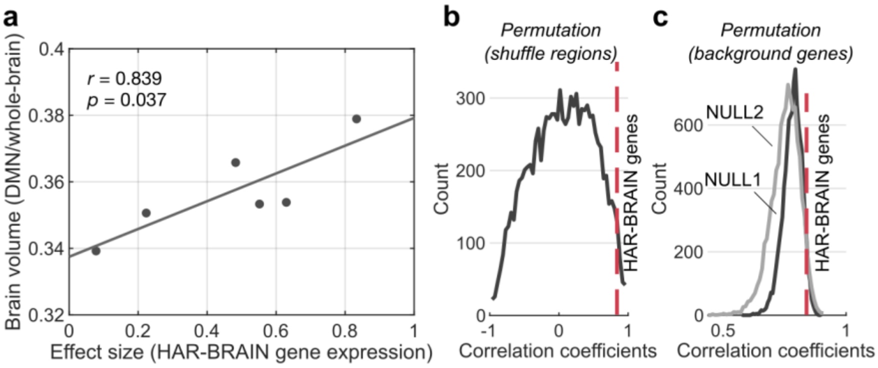

Supplementary Figure 4. *HAR-BRAIN gene expression and cortical volume*. (a) Effect size of HAR-BRAIN gene expression difference between the DMN and the rest of the cortex significantly correlates to the ratio of brain volume of the DMN regions (r = 0.839, p = 0.037). Brain volume is calculated by running FreeSurfer on the *ex-vivo* T1-weighted MRI of each brain donor (downloaded from https://human.brain-map.org/mri_viewers/data). (b) The correlation coefficient in (a) significantly exceeds the null distribution of correlation coefficients generated by randomly shuffling brain regions (two-sided p = 0.047, 10,000 permutations). (c) The correlation coefficient in (a) does not significantly exceed the null distribution of correlation coefficients generated by randomly selecting background genes, with only trend-level effects observed for BRAIN genes (NULL1, p = 0.095, n.s.) and ECE genes (NULL2, p = 0.090, n.s., 10,000 permutations). Source data are provided as a Source Data file.

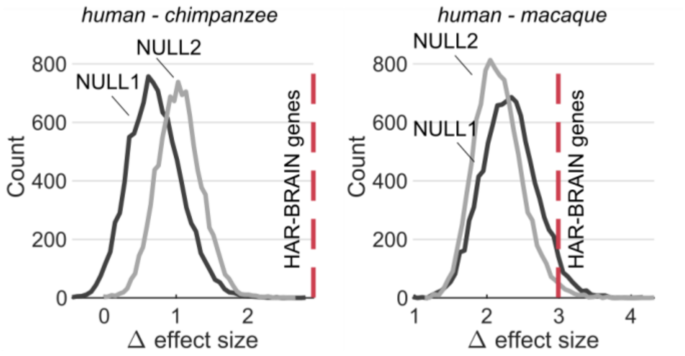

Supplementary Figure 5. *HAR-BRAIN gene expression enhancement in cognitive network regions in humans compared to chimpanzees/macaques.* Left: permutation testing shows the Δ effect size of the enhanced HAR-BRAIN gene expression in cognitive network regions between humans and chimpanzees to significantly exceed null distributions of Δ effect size computed by randomly selecting gene sets from the pool of BRAIN genes (NULL1: $p < 0.001$) and evolutionarily conserved genes (ECE genes; NULL2: $p < 0.001$, 10,000 permutations). Right: permutation testing shows the Δ effect size of the enhanced HAR-BRAIN gene expression in cognitive network regions between humans and macaques to significantly exceed NULL2 (two-sided $p = 0.026$), but not NULL1 (two-sided $p = 0.090$, 10,000 permutations). The marginal trend-level effect found for NULL1 might be due to the notion of macaques and humans to be more genetically different as compared to chimpanzees, and the set of HAR-BRAIN genes thus to only partially cover the evolutionarily genetic differentiations between the human and macaque and many genetically differences to remain in the NULL condition.

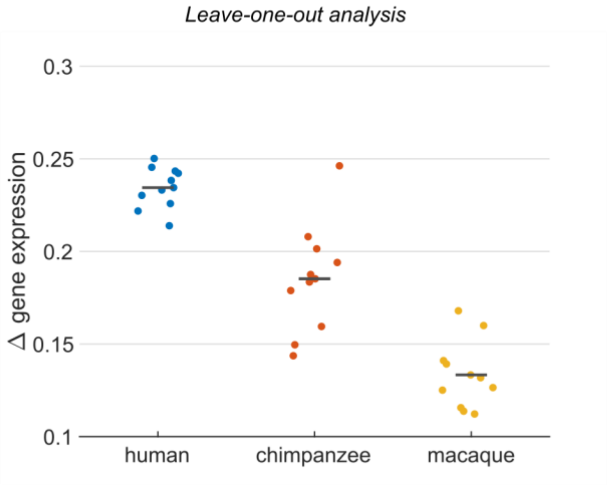

Supplementary Figure 6. Leave-one-out analysis by computing the difference of mean gene expression (Δ gene expression) between cognitive network regions and primary regions for ten rounds, in each of which one region out of the ten regions was excluded. The resulting Δ gene expression in humans is larger than that in chimpanzees in 9/10 rounds and that in macaques in all 10 rounds.

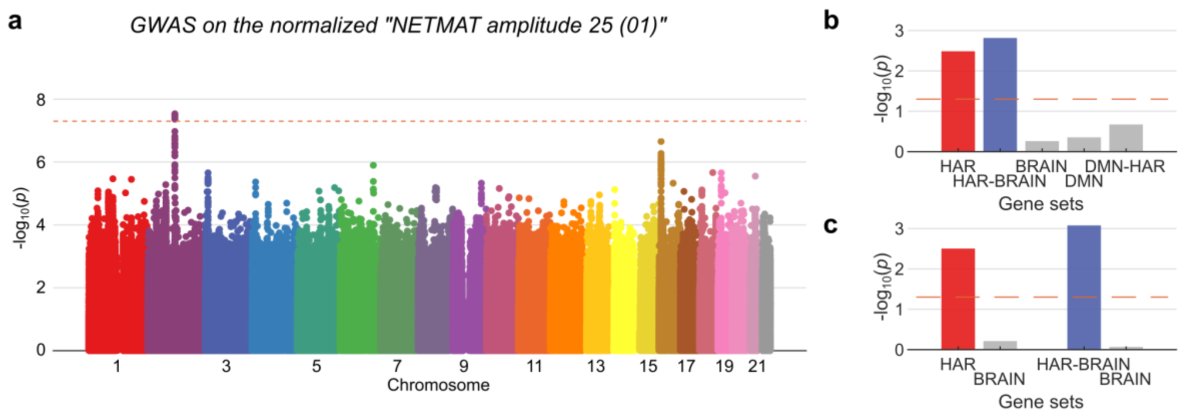

Supplementary Figure 7. *GWAS on the normalized DMN functional activity*. (a) Manhattan plot showing $-\log_{10}$-transformed two-tailed p value of each SNP from the GWAS analysis on the y axis and base-pair positions along the chromosomes on the x axis. Dotted red line indicates Bonferroni-corrected genome-wide significance ($p < 5 \times 10^{-8}$). (b) MAGMA gene-set analysis. $-\log_{10}$-transformed p-values of the associations between HAR, HAR-BRAIN, BRAIN, DMN, and DMN-HAR genes and DMN functional activity are displayed. Dashed line indicates $p = 0.05$. (c) MAGMA conditional gene-set analysis. $-\log_{10}$-transformed p-values of the associations between HAR and HAR-BRAIN genes and DMN functional activity are displayed, with BRAIN genes taken as covariant. Dashed line indicates $p = 0.05$.

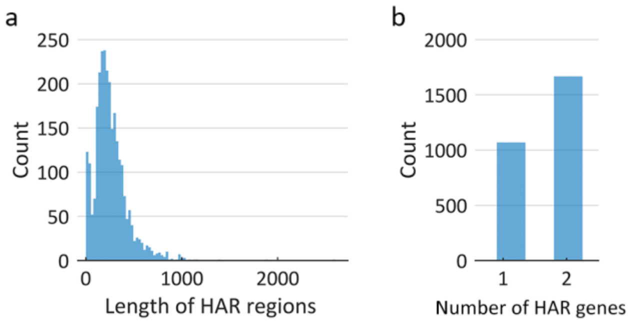

Supplementary Figure 8. Distributions of lengths of the HAR regions (a) and the number of genes mapped from each HAR region (b).

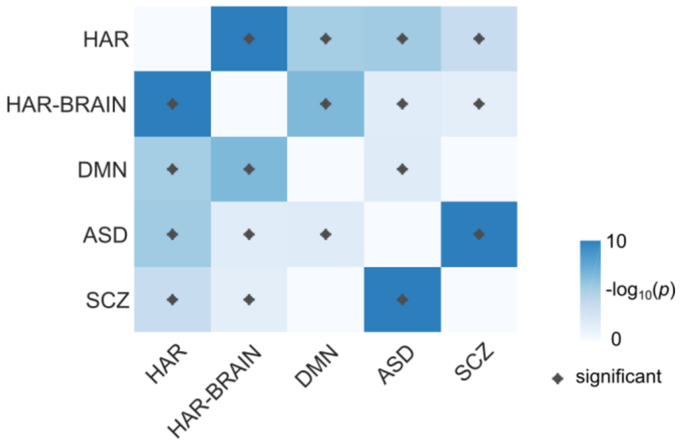

Supplementary Figure 9. Enrichment of HAR, HAR-BRAIN, and DMN genes in rare variations of ASD and schizophrenia (SCZ). *P*-values obtained from hypergeometric test.

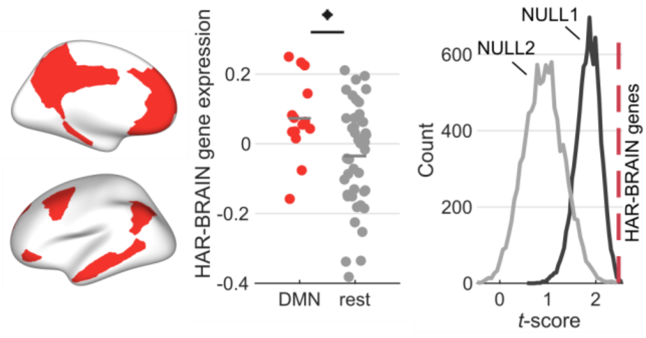

Supplementary Figure 10. *DMN assignment adapted from Raichle (2015).* Using the DMN assignment adapted from Raichle (2015) (left panel), DMN regions consistently show higher gene expression of HAR-BRAIN genes compared to the rest of the cortex ($t(55) = 2.476$, $p = 0.016$) (middle panel), with the t-score significantly exceeding null distributions generated by random BRAIN genes (NULL1, $p = 0.003$) and ECE genes (NULL2, $p < 0.001$) (right panel).

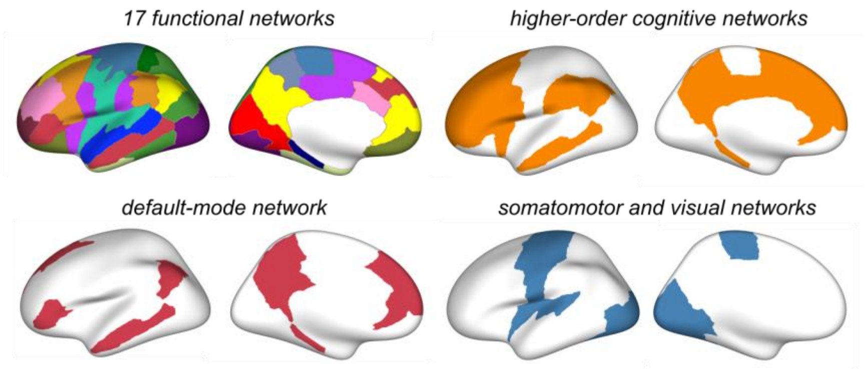

Supplementary Figure 11. *Seventeen functional networks.* DK cortical regions were assigned to 17 functional networks for validation (top left panel). Networks labeled as 11, 15, 16, and 17 were grouped into the default-mode network (bottom left panel). Grouping the default-mode network and networks labeled as 7, 8, 12, and 13 formed the higher-order cognitive networks (top right panel). Networks labeled as 1, 2, 3, and 4 were defined as primary networks (bottom right panel).

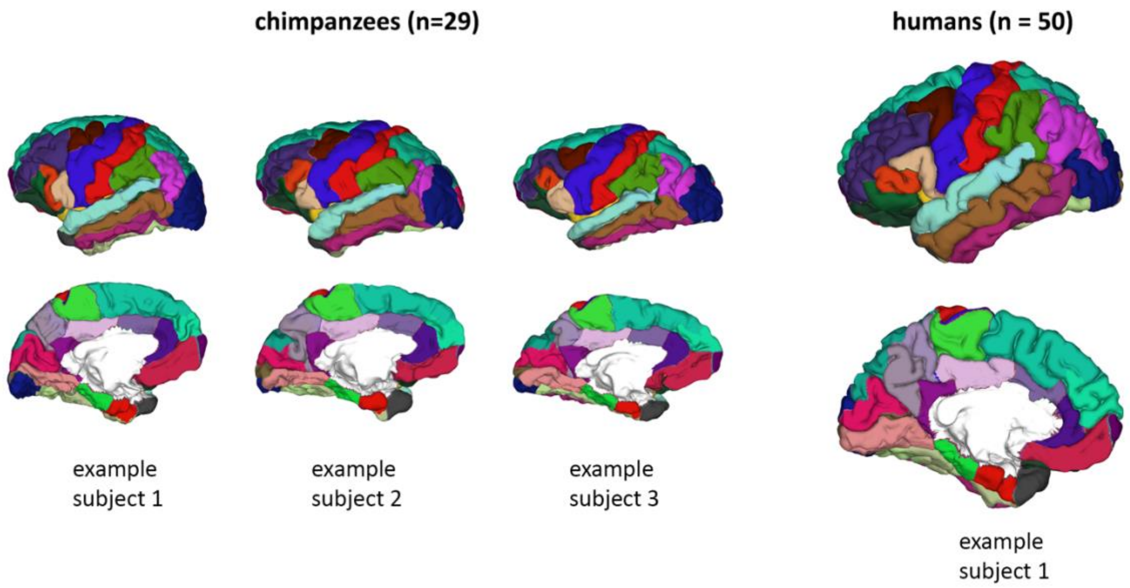

Supplementary Figure 12. *Example cortical parcellations of chimpanzees and humans.*

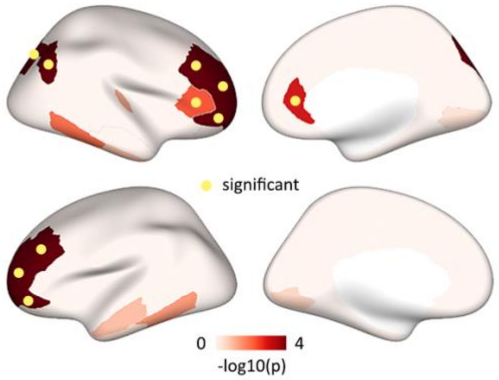

Supplementary Figure 13. Brain maps of regions showing significant chimpanzee-to-human expansion in permutation testing based on region shuffling. *P*-values obtained from 10,000 permutations.

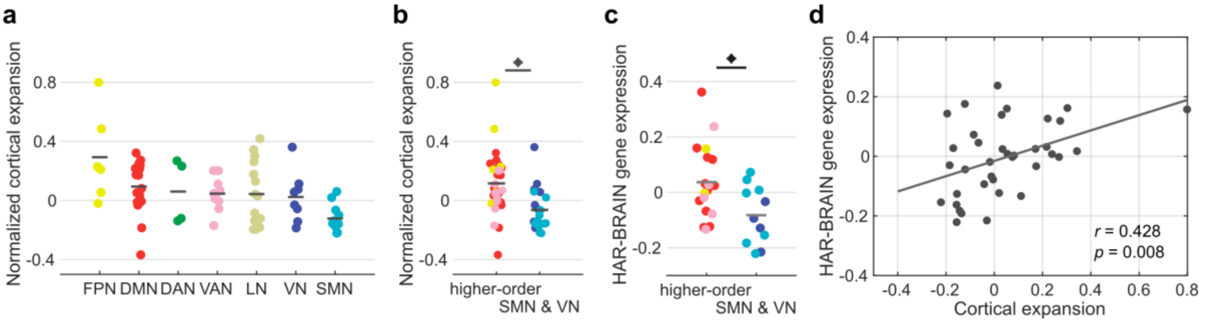

Supplementary Figure 14. *Evolutionary cortical expansion and HAR-BRAIN gene expression in BB-38 chimpanzee-human atlas*. (a) Levels of the cortical expansion per functional network in descending order. VN: visual network, SMN: somatomotor network, DAN: dorsal-attention network, LN: limbic network, VAN: ventral-attention network, FPN: frontal parietal network, DMN: default-mode network. (b) Regions of higher-order cognitive networks (DMN, FPN, VAN), present significant larger expansion compared to regions in SMN and VN ($t(28) = 3.632$, $p < 0.001$). (c) Regions of higher-order cognitive networks (DMN, FPN, VAN), show significantly higher HAR-BRAIN expression compared to regions in SMN and VN ($t(28) = 2.572$, $p = 0.016$). (d) Cortical expansion significantly correlates to cortical HAR-BRAIN gene expression ($r = 0.428$, $p = 0.008$). Source data are provided as a Source Data file.

Supplementary References

1. Li L, *et al.* Mapping putative hubs in human, chimpanzee and rhesus macaque connectomes via diffusion tractography. *Neuroimage* **80**, 462-474 (2013).

2. Van Essen DC, *et al.* The WU-Minn Human Connectome Project: an overview. *Neuroimage* **80**, 62-79 (2013).

3. Fischl B. FreeSurfer. *Neuroimage* **62**, 774-781 (2012).

4. Fischl B, *et al.* Automatically parcellating the human cerebral cortex. *Cereb Cortex* **14**, 11-22 (2004).

5. Donahue CJ, Glasser MF, Preuss TM, Rilling JK, Van Essen DC. Quantitative assessment of prefrontal cortex in humans relative to nonhuman primates. *Proc Natl Acad Sci USA* **115**, E5183-E5192 (2018).

6. Desikan RS, *et al.* An automated labeling system for subdividing the human cerebral cortex on MRI scans into gyral based regions of interest. *Neuroimage* **31**, 968-980 (2006).

7. Cammoun L, *et al.* Mapping the human connectome at multiple scales with diffusion spectrum MRI. *J Neurosci Methods* **203**, 386-397 (2012).

8. Scholtens LH, de Reus MA, de Lange SC, Schmidt R, van den Heuvel MP. An MRI Von Economo - Koskinas atlas. *Neuroimage*, (2016).

9. van den Heuvel MP, *et al.* Evolutionarily developed connections compromised in schizophrenia. Preprint at https://www.biorxiv.org/content/10.1101/387506v1 (2018).

10. Bailey P, Bonin GV, McCulloch WS. *The isocortex of the chimpanzee*. Univ. Illinois Press (1950).

11. von Economo CF, Koskinas GN. *Die cytoarchitektonik der hirnrinde des erwachsenen menschen*. Julius Springer Verlag (1925).

12. Hawrylycz MJ, *et al.* An anatomically comprehensive atlas of the adult human brain transcriptome. *Nature* **489**, 391-399 (2012).

13. Romme IA, de Reus MA, Ophoff RA, Kahn RS, van den Heuvel MP. Connectome Disconnectivity and Cortical Gene Expression in Patients With Schizophrenia. *Biol Psychiatry* **81**, 495-502 (2017).

14. French L, Paus T. A FreeSurfer view of the cortical transcriptome generated from the Allen Human Brain Atlas. *Front Neurosci* **9**, 323 (2015).

15. Sousa AMM, *et al.* Molecular and cellular reorganization of neural circuits in the human lineage. *Science* **358**, 1027-1032 (2017).

16. Team RC. R: A language and environment for statistical computing. (2013).

17. Arnatkevičiūtė A, Fulcher BD, Fornito A. A practical guide to linking brain-wide gene expression and neuroimaging data. *Neuroimage*, (2019).

18. Doan RN, *et al.* Mutations in Human Accelerated Regions Disrupt Cognition and Social Behavior. *Cell* **167**, 341-354 e312 (2016).

19. Wang DF, *et al.* Comprehensive functional genomic resource and integrative model for the human brain. *Science* **362**, 1266-+ (2018).

20. Yeo BT, *et al.* The organization of the human cerebral cortex estimated by intrinsic functional connectivity. *J Neurophysiol* **106**, 1125-1165 (2011).

21. Raichle ME. The brain's default mode network. *Annu Rev Neurosci* **38**, 433-447 (2015).

22. Gao XY, Stamier J, Martin ER. A multiple testing correction method for genetic association studies using correlated single nucleotide polymorphisms. *Genet Epidemiol* **32**, 361-369 (2008).

23. Shriner D, Baye TM, Padilla MA, Zhang S, Vaughan LK, Loraine AE. Commonality of functional annotation: a method for prioritization of candidate genes from genome-wide linkage studies. *Nucleic Acids Research* **36**, (2008).

24. Li J, Ji L. Adjusting multiple testing in multilocus analyses using the eigenvalues of a correlation matrix. *Heredity* **95**, 221-227 (2005).

25. Ashburner M, *et al.* Gene ontology: tool for the unification of biology. The Gene Ontology Consortium. *Nat Genet* **25**, 25-29 (2000).

26. Sudlow C, *et al.* UK Biobank: An Open Access Resource for Identifying the Causes of a Wide Range of Complex Diseases of Middle and Old Age. *Plos Med* **12**, (2015).

27. Savage JE, *et al.* Genome-wide association meta-analysis in 269,867 individuals identifies new genetic and functional links to intelligence. *Nat Genet* **50**, 912-919 (2018).

28. Bycroft C, *et al.* The UK Biobank resource with deep phenotyping and genomic data. *Nature* **562**, 203-209 (2018).

29. McCarthy S, *et al.* A reference panel of 64,976 haplotypes for genotype imputation. *Nat Genet* **48**, 1279-1283 (2016).

30. Genomes Project C, *et al.* A global reference for human genetic variation. *Nature* **526**, 68-74 (2015).

31. Webb BT, *et al.* Molecular Genetic Influences on Normative and Problematic Alcohol Use in a Population-Based Sample of College Students. *Front Genet* **8**, 30 (2017).

32. Abraham G, Qiu Y, Inouye M. FlashPCA2: principal component analysis of Biobank-scale genotype datasets. *Bioinformatics* **33**, 2776-2778 (2017).

33. Elliott LT, *et al.* Genome-wide association studies of brain imaging phenotypes in UK Biobank. *Nature* **562**, 210-216 (2018).

34. Miller KL, *et al.* Multimodal population brain imaging in the UK Biobank prospective epidemiological study. *Nat Neurosci* **19**, 1523-1536 (2016).

35. Purcell S, *et al.* PLINK: a tool set for whole-genome association and population-based linkage analyses. *The American Journal of Human Genetics* **81**, 559-575 (2007).

36. Watanabe K, Taskesen E, van Bochoven A, Posthuma D. Functional mapping and annotation of genetic associations with FUMA. *Nat Commun* **8**, 1826 (2017).

37. Wang K, Li MY, Hakonarson H. ANNOVAR: functional annotation of genetic variants from high-throughput sequencing data. *Nucleic Acids Research* **38**, (2010).

38. Ardlie KG, *et al.* The Genotype-Tissue Expression (GTEx) pilot analysis: Multitissue gene regulation in humans. *Science* **348**, 648-660 (2015).

39. Westra HJ, *et al.* Systematic identification of trans eQTLs as putative drivers of known disease associations. *Nature Genetics* **45**, 1238-U1195 (2013).

40. Zhernakova DV, *et al.* Identification of context-dependent expression quantitative trait loci in whole blood. *Nature Genetics* **49**, 139-145 (2017).

41. Wang D, *et al.* Comprehensive functional genomic resource and integrative model for the human brain. *Science* **362**, (2018).

42. Schmitt AD, *et al.* A Compendium of Chromatin Contact Maps Reveals Spatially Active Regions in the Human Genome. *Cell Rep* **17**, 2042-2059 (2016).

43. Kundaje A, *et al.* Integrative analysis of 111 reference human epigenomes. *Nature* **518**, 317-330 (2015).

44. MacArthur J, *et al.* The new NHGRI-EBI Catalog of published genome-wide association studies (GWAS Catalog). *Nucleic Acids Research* **45**, D896-D901 (2017).

45. de Leeuw CA, Mooij JM, Heskes T, Posthuma D. MAGMA: generalized gene-set analysis of GWAS data. *PLoS Comput Biol* **11**, e1004219 (2015).

46. Fox PT, Lancaster JL. Mapping context and content: the BrainMap model. *Nature Reviews Neuroscience* **3**, 319-321 (2002).

47. Fox PT, *et al.* BrainMap taxonomy of experimental design: description and evaluation. *Hum Brain Mapp* **25**, 185-198 (2005).

48. Vanasse TJ, *et al.* BrainMap VBM: An environment for structural meta-analysis. *Hum Brain Mapp*, (2018).

49. Eickhoff SB, Laird AR, Grefkes C, Wang LE, Zilles K, Fox PT. Coordinate-based activation likelihood estimation meta-analysis of neuroimaging data: a random-effects approach based on empirical estimates of spatial uncertainty. *Hum Brain Mapp* **30**, 2907-2926 (2009).

50. Turkeltaub PE, Eickhoff SB, Laird AR, Fox M, Wiener M, Fox P. Minimizing within-experiment and within-group effects in Activation Likelihood Estimation meta-analyses. *Hum Brain Mapp* **33**, 1-13 (2012).

51. Donahue CJ, Glasser MF, Preuss TM, Rilling JK, Van Essen DC. Quantitative assessment of prefrontal cortex in humans relative to nonhuman primates. *Proc Natl Acad Sci USA* **115**, E5183-E5192 (2018).

52. Hill J, Inder T, Neil J, Dierker D, Harwell J, Van Essen D. Similar patterns of cortical expansion during human development and evolution. *Proc Natl Acad Sci USA* **107**, 13135-13140 (2010).

53. MacLean PD. *The triune brain in evolution: Role in paleocerebral functions*. Springer Science & Business Media (1990).

54. Mechelli A, Humphreys GW, Mayall K, Olson A, Price CJ. Differential effects of word length and visual contrast in the fusiform and lingual gyri during reading. *Proc Biol Sci* **267**, 1909-1913 (2000).
