# Supplementary material for: Genetic mapping and evolutionary analysis of human-expanded cognitive networks
Source: Nat Commun. 2019 Oct 24;10:4839. doi: 10.1038/s41467-019-12764-8 (PMC6813316; doi:10.1038/s41467-019-12764-8)
Supplement: Supplementary file 3 — Description of Additional Supplementary Files [file 41467_2019_12764_MOESM3_ESM.pdf]

**Description of Additional Supplementary Files**

File Name: Supplementary Data 1
Description: Cortical surface area expansion between the chimpanzee and human.

File Name: Supplementary Data 2
Description: List of HAR and HAR-BRAIN genes.

File Name: Supplementary Data 3
Description:  List of DMN genes.
