# Supplementary material for: Genetic mapping and evolutionary analysis of human-expanded cognitive networks
Source: Nat Commun. 2019 Oct 24;10:4839. doi: 10.1038/s41467-019-12764-8 (PMC6813316; doi:10.1038/s41467-019-12764-8)
Supplement: Supplementary file 7 — Reporting Summary [file 41467_2019_12764_MOESM7_ESM.pdf]

nature research

Corresponding author(s): M.P. van den Heuvel

Last updated by author(s): Aug 14, 2019

Reporting Summary

Nature Research wishes to improve the reproducibility of the work that we publish. This form provides structure for consistency and transparency in reporting. For further information on Nature Research policies, see Authors & Referees and the Editorial Policy Checklist.

Statistics

For all statistical analyses, confirm that the following items are present in the figure legend, table legend, main text, or Methods section.

n/a	Confirmed	
☐	☒	The exact sample size (*n*) for each experimental group/condition, given as a discrete number and unit of measurement
☐	☒	A statement on whether measurements were taken from distinct samples or whether the same sample was measured repeatedly
☐	☒	The statistical test(s) used AND whether they are one- or two-sided<br>*Only common tests should be described solely by name; describe more complex techniques in the Methods section.*
☐	☒	A description of all covariates tested
☐	☒	A description of any assumptions or corrections, such as tests of normality and adjustment for multiple comparisons
☐	☒	A full description of the statistical parameters including central tendency (e.g. means) or other basic estimates (e.g. regression coefficient) AND variation (e.g. standard deviation) or associated estimates of uncertainty (e.g. confidence intervals)
☐	☒	For null hypothesis testing, the test statistic (e.g. *F*, *t*, *r*) with confidence intervals, effect sizes, degrees of freedom and *P* value noted<br>*Give P values as exact values whenever suitable.*
☒	☐	For Bayesian analysis, information on the choice of priors and Markov chain Monte Carlo settings
☒	☐	For hierarchical and complex designs, identification of the appropriate level for tests and full reporting of outcomes
☐	☒	Estimates of effect sizes (e.g. Cohen's *d*, Pearson's *r*), indicating how they were calculated

Our web collection on statistics for biologists contains articles on many of the points above.

Software and code

Policy information about availability of computer code

Data collection	Data from the BrainMap database were collected using the Sleuth toolbox (version 2.4; http://www.brainmap.org/sleuth).
Data analysis	The MRI data were analyzed using FreeSurfer (v5.3.0; http://surfer.nmr.mgh.harvard.edu) and FSL (v5.0, http://fsl.fmrib.ox.ac.uk).<br>The Genotype data were analyzed using PLINK (v2.0; https://cog-genomics.org/plick2).<br>Gene annotation was performed using FUMA (https://fuma.ctglab.nl).<br>Gene-set analysis was performed using MAGMA (v1.07; https://ctg.cncr.nl/software/magma).<br>All other data were analyzed using custom-written MATLAB code, based on MATLAB R2018a (MathWorks, Natick, USA).

For manuscripts utilizing custom algorithms or software that are central to the research but not yet described in published literature, software must be made available to editors/reviewers. We strongly encourage code deposition in a community repository (e.g. GitHub). See the Nature Research guidelines for submitting code & software for further information.

Data

Policy information about availability of data

All manuscripts must include a data availability statement. This statement should provide the following information, where applicable:

- Accession codes, unique identifiers, or web links for publicly available datasets
- A list of figures that have associated raw data
- A description of any restrictions on data availability

The human gene expression data that support the findings of this study are available in the Allen Brain Atlas (http://human.brain-map.org).
The comparative gene expression data that support the findings of this study are available in the PsychENCODE (http://evolution.psychencode.org).
The human MRI data (in the part of the cortical expansion) that support the findings of this study are available from the Human Connectome Project (https://www.humanconnectome.org).
The chimpanzee MRI data that support the findings of this study are available as part of the National Chimpanzee Brain Resources (www.chimpanzeebrain.org).
The genotype data (in the GWAS part) that support the findings of this study are available in the UK Biobank (application 16406; https://www.ukbiobank.ac.uk).

The GWAS summary statistics for "intelligence" that support the findings of this study are available from the lab's website (https://ctg.cncr.nl/software/summary_statistics).
The GWAS summary statistics for "schizophrenia" that support the findings of this study are available from the Psychiatric Genomics Consortium (http://www.med.unc.edu/pgc).
The GWAS summary statistics for ''Frequency of friend/family visits' that support the findings of this study are available from the GWAS ATLAS webtool (http://atlas.ctg.nl/traitDB/3216).
The cross-disorder VBM data that support the findings of this study are available in the BrainMap (http://www.brainmap.org).

Field-specific reporting

Please select the one below that is the best fit for your research. If you are not sure, read the appropriate sections before making your selection.

☒ Life sciences ☐ Behavioural & social sciences ☐ Ecological, evolutionary & environmental sciences

For a reference copy of the document with all sections, see nature.com/documents/nr-reporting-summary-flat.pdf

Life sciences study design

All studies must disclose on these points even when the disclosure is negative.

Sample size	Sample size of the human MRI data was determined by matching the available chimpanzee MRI data. Sample size of other data was based on data availability. No statistical methods were used to pre-determine sample size.
Data exclusions	For the genotype data, all files were checked for data integrity and accuracy. SNPs were filtered from further analysis if they met any of the following criteria: imputation quality (INFO/R2) score < 0.6, Hardy–Weinberg equilibrium p < 5e–6, minor allele frequency (MAF) < 0.005, and mismatch of alleles or allele frequency difference greater than 20% from the Haplotype HRC genome reference panel. Indels and SNPs that were duplicated, multiallelic, monomorphic, or ambiguous (A/T or C/G with MAF > 0.4) were also excluded (see Supplementary Methods).<br><br>For the MRI data from chimpanzees, five of the 29 chimpanzees were excluded as FreeSurfer failed to properly parcellate the parahippocampal gyrus and entorhinal cortex.
Replication	The main results were validated by using several alternative anatomical parcellation atlases and several different functional network divisions, by directly mapping the ~400 tissue samples for each donor in AHBA dataset to each of the functional networks, and by re-analyzing our data with various parameter settings, which all revealed consistent results (for details, see Supplementary Results)
Randomization	NA. Samples were not allocated into groups.
Blinding	Blinding was not relevant to our study as data were obtained from previous studies and open datasets.

Reporting for specific materials, systems and methods

We require information from authors about some types of materials, experimental systems and methods used in many studies. Here, indicate whether each material, system or method listed is relevant to your study. If you are not sure if a list item applies to your research, read the appropriate section before selecting a response.

Materials & experimental systems

n/a	Involved in the study	
☒	☐	Antibodies
☒	☐	Eukaryotic cell lines
☒	☐	Palaeontology
☐	☒	Animals and other organisms
☐	☒	Human research participants
☒	☐	Clinical data

Methods

n/a	Involved in the study	
☒	☐	ChIP-seq
☒	☐	Flow cytometry
☐	☒	MRI-based neuroimaging

Animals and other organisms

Policy information about studies involving animals; ARRIVE guidelines recommended for reporting animal research

Laboratory animals	Chimpanzees (Pan troglodytes), mean age ± standard deviation: 30.2 ± 12.6 years, all female.
Wild animals	No wild animals were used in this study.
Field-collected samples	No field-collected samples were used in this study.
Ethics oversight	All procedures were implemented under protocols approved by the YNPRC and the Emory University Institutional Animal Care

Ethics oversight	and Use Committee (IACUC, approval #: YER-2001206).

Note that full information on the approval of the study protocol must also be provided in the manuscript.

Human research participants

Policy information about studies involving human research participants

Population characteristics	We utilized data collected previously by external sources.
Recruitment	We utilized data collected previously by external sources.
Ethics oversight	We utilized data collected previously by external sources. All individuals included in the study provided informed consent. All original studies were approved by the concerned ethical committee.

Note that full information on the approval of the study protocol must also be provided in the manuscript.

Magnetic resonance imaging

Experimental design

Design type	NA
Design specifications	NA
Behavioral performance measures	NA

Acquisition

Imaging type(s)	Structural MRI
Field strength	3 Tesla
Sequence & imaging parameters	T1-weighted MRI from chimpanzees: double spin echo EPI, slice thickness = 0.8 mm, voxel size = 0.8 × 0.8 × 0.8 mm3, TR = 2,600 ms, TE = 3.06 ms, matrix size = 256 × 256 × 192, FOV = 224 × 224, flip angle = 8 degree, scanning time = 16 min.<br><br>Detailed descriptions of the MRI data from the Human Connectome Project are available at: https://www.humanconnectome.org/study/hcp-young-adult/document/q3-data-release
Area of acquisition	Whole brain

Diffusion MRI ☐ Used ☒ Not used

Preprocessing

Preprocessing software	T1-weighted images: Freesurfer v5.3.0
Normalization	NA
Normalization template	NA
Noise and artifact removal	NA
Volume censoring	NA

Statistical modeling & inference

Model type and settings	NA.
Effect(s) tested	NA

Specify type of analysis: ☐ Whole brain ☐ ROI-based ☒ Both

Anatomical location(s)	Desikan-Killiany atlas (Desikan et al., 2016).
Statistic type for inference (See Eklund et al. 2016)	NA
Correction	NA

Models & analysis

n/a	Involved in the study
☒	☐ Functional and/or effective connectivity
☒	☐ Graph analysis
☒	☐ Multivariate modeling or predictive analysis
